# Microsecond Dynamics of Fc–CD16a Recognition: Impact of Mutations, Core Fucosylation, and Fc Asymmetry

**DOI:** 10.3390/antib15010017

**Published:** 2026-02-23

**Authors:** Sébastien Estaran, Bernard Hehlen, Alain Chavanieu

**Affiliations:** 1 Institut des Biomolécules Max Mousseron (IBMM), Université de Montpellier, CNRS, École Nationale Supérieure de Chimie de Montpellier (ENSCM), 34090 Montpellier, France; 2 Laboratoire Charles Coulomb, UMR 5221, CNRS, Université de Montpellier, 34095 Montpellier, France

**Keywords:** Fc-FcγRIIIa interaction, antibody-dependent cellular cytotoxicity (ADCC), molecular dynamics simulation, non-covalent interactions, DFTE mutations, fucosylation, protein–glycan interaction

## Abstract

Background/Objectives: Antibody-dependent cellular cytotoxicity relies on the interaction between the Fc region of immunoglobulin G1 (IgG1) and the CD16a receptor. While removal of core fucosylation on Fc and introduction of the DFTE mutation set (S239D, H268F, S324T, I332E) are known to enhance CD16a binding, the detailed contributions of these engineered sites in solution remain incompletely defined. Methods: Here, we employed 1 µs molecular dynamics simulations to map, at atomic resolution, the interaction networks stabilizing pre-formed Fc-CD16a complexes, including afucosylated Fc-wild-type, DFTE-engineered, Fc-fucosylated, and asymmetrically engineered Fc variants. Results: Our results show that only S239D, present on both Fc chains, and H268F on chain A consistently contribute to stabilizing the CD16a interface, while I332E does not form persistent interactions. Glycan–protein contacts are primarily intrachain, with transient interchain glycan–glycan interactions not contributing significantly to complex stability. Fucosylation on Fc significantly reduces binding stability by disrupting peripheral interactions and critical glycan-mediated contacts. Notably, the asymmetric Fc variant, in which the two heavy chains carry distinct sets of substitutions, retains high-affinity binding despite lacking S239D and carrying core fucose, through a novel hydrophobic cluster and reinforced peripheral electrostatic interactions. Conclusions: Altogether, these findings provide a quantitative framework for how targeted mutations and fucose modifications remodel Fc-CD16a interactions, offering insights for the rational design of next-generation therapeutic antibodies.

## 1. Introduction

Immunoglobulins, commonly known as antibodies, are essential components of the immune system. These Y-shaped molecules, composed of two heavy and two light chains, contain two fragment antigen-binding (Fab) domains that confer antigen specificity and have significant therapeutic applications, such as blocking pathogen adherence to host cells [1,2,3,4]. The Fab regions are connected via a flexible hinge to the fragment crystallizable (Fc) domain, which is crucial for immune activation. Beyond maintaining structural integrity, the Fc region interacts with various Fc receptors on immune cells, playing a pivotal role in modulating immune responses [5].

Among the Fc receptors, Fc gamma receptors (FcγRs) mediate key immune functions by interacting with the Fc region of IgG antibodies. These receptors facilitate a variety of cellular processes including antibody-dependent cellular cytotoxicity (ADCC) by NK cells, antibody-dependent cellular phagocytosis (ADCP) by macrophages and monocytes, and degranulation by eosinophils and basophils [6]. Additionally, the Fc region can initiate the complement cascade via the classical pathway, further enhancing immune activation and effector cell recruitment [7]. Given the diversity of FcγR-mediated functions, precisely modulating Fc-FcγR interactions presents significant therapeutic potential for optimizing immune responses [8,9].

In humans, the FcγR family consists of six distinct receptors with varying affinities for IgG, FcγRI (CD64), FcγRIIa (CD32a), FcγRIIb (CD32b), FcγRIIc (CD32c), FcγRIIIa (CD16a), and FcγRIIIb (CD16b) [6,9]. FcγRI is the only Fcγ receptor with high affinity for monomeric IgG, whereas the other FcγRs bind monomeric IgG with lower affinities and are functionally engaged predominantly in the context of immune complexes. Functionally, FcγRs are categorized into activatory and inhibitory receptors. Activatory Fcγ receptors (FcγRI, FcγRIIa, FcγRIIc, and FcγRIIIa) signal through immunoreceptor tyrosine-based activation motifs (ITAMs), either embedded in their cytoplasmic domains or provided by associated signaling chains, while FcγRIIb, the sole inhibitory receptor, possesses an immunoreceptor tyrosine-based inhibitory motif (ITIM) that is crucial for dampening immune responses and maintaining immune homeostasis. FcγRIIIb, a GPI-anchored receptor, lacks ITAM/ITIM domains but modulates neutrophil functions [10]. The binding affinities of different IgG subclasses further shape these dynamics, with hIgG1 and hIgG3 exhibiting the highest affinities for FcγRs, whereas hIgG4 binds more weakly [10,11].

Therapeutic antibodies have revolutionized biopharmaceuticals, with CD16a playing a central role in ADCC-mediated tumor clearance [12]. Natural killer (NK) cells, which predominantly express CD16a, recognize Fc regions of antibodies bound to tumor cells, triggering cytotoxic granule release and subsequent tumor lysis [13,14]. Notable examples include rituximab [15], trastuzumab [16], and cetuximab [17,18], which have demonstrated significant NK cell-mediated ADCC in vitro.

Over the past decades, efforts have been devoted to enhancing IgG-CD16a interactions to optimize ADCC-based therapies [2,8,19,20,21]. The primary CD16a contact interface involves the B/C loop (Trp113-Ala117), F/G loop (Val158-Lys161), C strand (His119-Thr122), and C9 strand (Lys131-His134). These regions engage asymmetrically with each Fc chain, involving the lower hinge, B strand, and multiple loop regions of Fc, specifically Asp265-Glu269, Asn297-Thr299, Ala327-Ile332, and the hinge region spanning Leu234-Ser239 [22,23,24,25].

Removal of core fucose from the Fc glycans significantly enhances CD16a binding, decreasing the KD from ~300–1000 nM to ~70–100 nM in afucosylated wild-type antibodies [24,26,27,28]. However, it is essential to acknowledge that absolute affinity values vary across studies due to methodological differences (e.g., SPR vs. ITC), glycoform heterogeneity, and, notably, the CD16a allotype employed [22,28,29,30,31]. The high-affinity variant V158 consistently exhibits stronger binding than the low-affinity F158 variant, often by a factor of 5 to 10.

The pioneering work of Shields et al. identified key residues within the lower hinge and the CH2 domain of Fc as critical for CD16a binding [11]. Notably, mutations such as S239D and I332E either alone or in combination within variants such as GASDALIE [19,32,33] (G236A/S239D/A330L/I332E) have been shown to significantly increase CD16a binding affinity into the nanomolar range. This enhanced affinity correlates with increased ADCC potency, making them key modifications in therapeutic antibodies like alemtuzumab and rituximab [2,8,26]. These mutations exemplify Fc engineering strategies aimed at optimizing Fc–receptor interactions and downstream immune activation [31,32,34]. More recently, asymmetric Fc engineering has introduced differential substitutions into each Fc chain. For instance, L234Y, G236W, S239M, H268D in one domain and D270E, A330M, K334E in the other, to refine receptor selectivity and tune signaling. This design concept represents a novel approach to decoupling binding strength from immune activation, and is further explored in the present study [22,30,35,36,37].

Structural studies and short molecular dynamics simulations have identified key stabilizing elements at the Fc-CD16a interface. On Fc chain A, Ser239 and Asp265 interact with Lys120 of CD16a, while on chain B, Asp265 forms a critical salt bridge with Lys161 [22,23,24,25,26]. Additional contacts include interactions between Glu233, Glu269 and CD16a residues Lys128 and Lys131, and van der Waals packing involving Leu234, Leu235, and Pro329 of Fc against Trp90 and Trp113 of CD16a. These cumulative interactions define a compact, asymmetrical binding surface centered on the C_H_2 domain, with distinct contributions from chains A and B fine-tuning the overall affinity.

While these studies established the structural and energetic bases of FcγR engagement, their molecular interpretation remained largely static, relying on crystallographic snapshots [23,24,32,35] or submicrosecond simulations not resolving long-timescale stabilization of engineered mutants [27,33,38,39,40]. Complementary studies expanded the picture by analyzing the architectural contribution of FcγRI’s D3 ectodomain to Fc engagement, mapping transient hotspot contacts at the IgG–FcγRIIIa interface in full-length simulations, and describing antigen to Fc allostery that promotes Fc-receptor engagement [41,42,43].

Here, using molecular dynamics (MD), we present a microsecond-scale dynamic atlas of Fc–CD16a recognition integrating the four-mutation DFTE variant (S239D/H268F/S324T/I332E), core fucosylation, and Fc-chain asymmetry. Multi-replica trajectories of preformed complexes enable a statistical dissection of hydrogen bond, ionic, and van der Waals networks across protein–protein, protein–glycan, and glycan–glycan layers. We focus on central anchoring and peripheral contacts to highlight the structural adaptability of the complex and its dynamic response to engineered changes. Enhanced variants strengthen interfacial networks through complementary mechanisms including electrostatic reinforcement in DFTE-like mutants and increased van der Waals stabilization in the asymmetric Fc. Our analysis confirms that N-glycans predominantly engage in intrachain interactions with their respective Fc domains, with minimal contribution from interchain glycan bridges. These insights refine the current understanding of CD16a recognition, link local interaction persistence to experimentally observed affinity shifts, and enable a dynamic and differential mapping of the interaction network at atomic resolution. Our study provides a robust framework for predicting and guiding future Fc engineering strategies, extending to rational antibody design and de novo protein scaffolds.

## 2. Experimental Procedures

All modeling, simulation, and analysis procedures are described in detail in the Supplementary Information (Section 1). Briefly, homology models of human CD16a (V158) in complex with IgG1 Fc were constructed to examine the effects of Fc mutations, fucosylation, and asymmetry on the interaction network. Four complexes were generated: the wild-type afucosylated Fc (Fc-af), the four-mutation afucosylated variant (Fc4m-af; S239D/H268F/S324T/I332E), the corresponding fucosylated form (Fc4m-f), and an asymmetric mutant carrying distinct substitutions on the two Fc chains (FcAs-f).

Each model was built from high-resolution structural template (PDB 3AY4, 3WN5) using MODELLER and CHARMM-GUI to ensure consistent glycan composition and disulfide bonding. All systems were simulated for 1 µs per replica (four replicas per complex) with explicit solvent and full glycosylation using the CHARMM36m force field within GROMACS 2022.2.

Trajectory analyses were performed with MDAnalysis, RING 2.0, and custom Python3.7.12 scripts to quantify protein–protein, protein–glycan, and glycan–glycan contacts over the final 900 ns of each simulation. Hydrogen bond, ionic, and van der Waals interactions were compared across complexes using two-sided Welch tests on per-replica means. Convergence, structural stability, and solvent exposure were validated by RMSD, RMSF, radius of gyration, and SASA metrics. Although solvent was modeled explicitly, water-mediated interactions were not mapped in this study. Then, pairwise residue interaction energies between the Fc fragment (Fc A and Fc B) and CD16a were evaluated using the molecular mechanics/generalized Born surface area (MM/GBSA) approach implemented in gmx_MMPBSA.

A full description of model construction, dynamics protocols, analysis of molecular dynamics trajectories, statistical workflow, and software environments is provided in the *Supplementary Data*.

## 3. Results

### 3.1. Molecular Dynamics and Residue Interactions and at the Fc-CD16a Binding Interface

**Figure 1:**
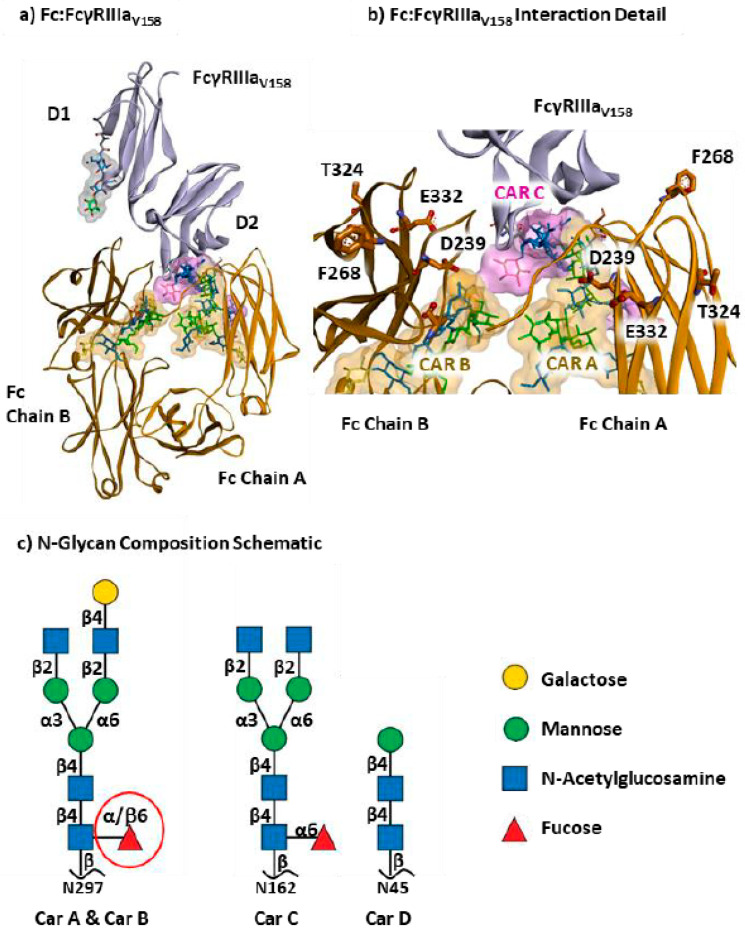
Structural representation of the Fc fragment in complex with CD16a. (**a**) Structure of the Fc fragment, chains A and B, in complex with CD16a (FcγRIIIaV158). The Fc chains are depicted in an orange ribbon representation, with their associated glycans, Car A and Car B, presented as surfaces and rendered as sticks to highlight their intricate structures. CD16a, with D1 and D2 domains, is shown as a ribbon in mauve, bearing two glycans represented as surfaces, Car C in pink and Car D in grey, and as sticks to elucidate their sugar compositions. The model here corresponds to the complex Fc4m-af with a Fc fragment bearing four mutations (DFTE) on each chain and an absence of fucose on Car A and Car B. For CD16a, the residue at position 158 is a valine. (**b**) Detailed visualization of the Fc-CD16a interaction interface illustrating the involvement of glycan chains Car A, Car B, and Car C, along with the key amino acid residues. For instance, the four mutations—D239, F268, T324, and E332—on each Fc chain are depicted as sticks. Four distinct complexes were analyzed during 1 μs molecular dynamics simulations. The Fc-af complex corresponds to a wild-type Fc lacking mutations and core fucosylation on glycan chains Car A and Car B, while the CD16a glycan (Car C) contains an α1–6-linked core fucose. The Fc4m-af and Fc4m-f complexes include four Fc mutations (S239D, H268F, S324T, I332E) symmetrically introduced on chains A and B. Fc4m-f additionally includes α1–6-linked core fucose on Fc glycan chains Car A and Car B (red circle). The asymmetrically engineered FcAs-f complex contains multiple mutations on Fc chain A (L234Y, L235Y, G236W, S239M, H268D, S298A, A327D) and Fc chain B (D270E, K326D, A330K, K334E, D356C, T366S, L368A, Y407V). In this system, the Fc core fucose on glycan chains Car A and Car B is β-linked, reflecting the stereochemistry present in the crystallographic template used for model construction (PDB ID: 3WN5). (**c**) Representation of N-glycan structures on the Fc and FcγRIIIaV158 complexes, depicted using the Symbol Nomenclature for Glycans (SNFG). The glycan chains on Car A and Car B were purposefully modeled without fucose in the Fc-af and Fc4m-af complexes prior to MD simulations. In contrast, these sites are α- and β-fucosylated in the Fc4m-f and FcAs-f complexes, respectively. Glycan chains are covalently attached to asparagine residues: N297 for Car A and Car B, N162 for Car C, and N45 for Car D.

The structure of the Fc fragment in complex with human CD16a (FcγRIIIaV158) is depicted in Figure 1, highlighting the involvement of both protein chains and three glycan chains in the interactions. Here, the Fc fragment comprises two identical heavy chains (A and B), each with four mutations: S239D, H268F, S324T, and I332E (DFTE), along with their associated afucosylated glycans, Car A and Car B at Asn-297. In this complex (Fc4m-af). CD16a carries a valine at position 158 (chain C) and two N-glycans, one on CAR C at Asn162 and one on CAR D at Asn45, and primarily engages the Fc fragment through its D2 domain, contacting both CH2 domains and the hinge region [23].

Combined, afucosylation [23,24,26,28] and DFTE mutations [11,44,45] act additively in modulating Fc-CD16a interactions, underscoring the complementary roles of glycan and sequence modifications To evaluate the impact of DFTE mutations and the presence of fucose on Car A and Car B on the interaction network between Fc and CD16a, three distinct protein complexes were engineered and subjected to 1 µs molecular dynamics simulations across four replicas. The systems included Fc-af, lacking both mutations and fucose; Fc4m-af, carrying four DFTE mutations without fucose; and Fc4m-f, which includes the same four mutations with fucose on both Fc glycans. In addition, we analyzed an asymmetric engineering Fc complex (FcAs-f), which also exhibits nanomolar affinity for CD16a [35,36]. This variant, featuring distinct mutations on Fc chains A and B, represents an alternative strategy for affinity optimization.

To assess how these modifications influence molecular interactions, hydrogen bonds and electrostatic contacts between proteins were first analyzed, followed by hydrogen bonds between proteins and glycans, and finally, intra- and inter-glycan hydrogen bonds over the final 900 ns of the simulations, excluding the initial 100 ns to allow for structural equilibration. In addition, van der Waals interactions between protein residues at the interfaces were examined to capture non-polar contributions to binding. All 16 μs-scale MD replicas reached stable backbones (per-chain RMSD ~1–3 Å) and compact complexes (Rg ~31–34 Å) with comparable residue-level flexibility across Fc variants and CD16a. Detailed analysis (RMSD/RMSF/Rg) is provided in Figures S1–S4. Per-residue SASA (Figure S5) was comparable across variants, and glycan C1-RMSF profiles converged at the GlcNAc1–GlcNAc2–Man3 core but diverged at distal antennae in a variant-dependent manner (Figure S6). The CAR B mobility is dampened by DFTE mutations. Fc core fucosylation modestly increases distal motion on CAR A and slightly agitates CD16a CAR C; detailed glycan dynamics are analyzed below.

### 3.2. Interactions Between the Fc Fragment and CD16a in Absence of Mutations and Fucose

#### 3.2.1. Protein to Protein Interactions

**Figure 2:**
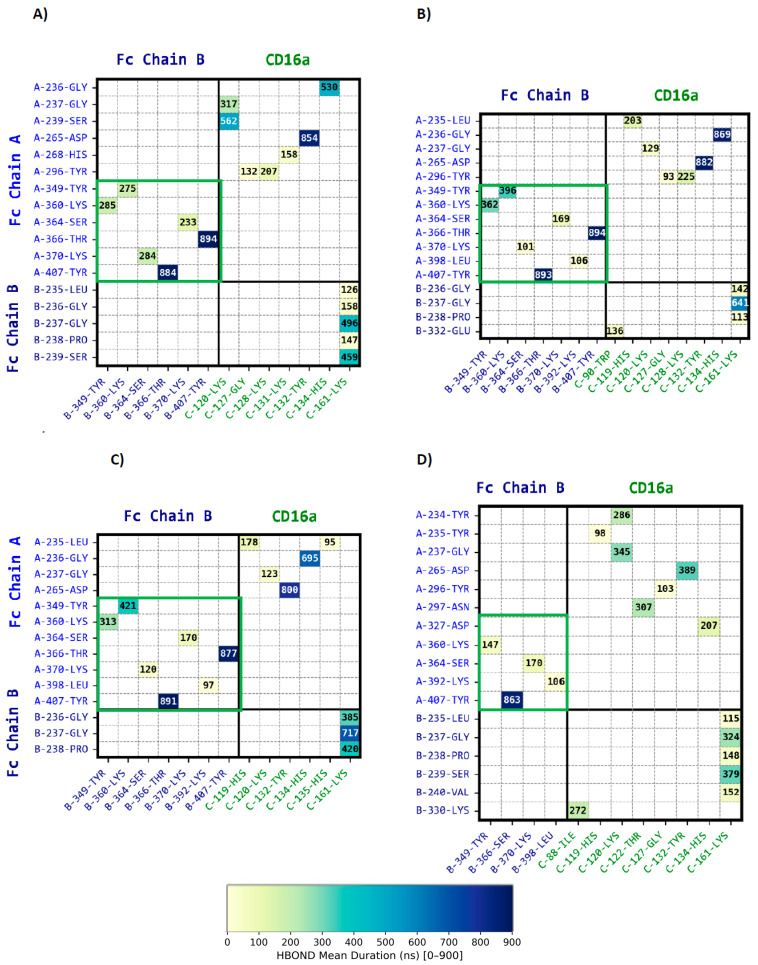
Heatmap representing intermolecular hydrogen bond interaction durations. The heatmap illustrates the average durations of hydrogen bond interactions at the inter-chain residue level over a 900 ns simulation. The analysis was performed within the Fc (Chains A and B) and CD16a across four distinct complexes: Fc-af (**A**), Fc4m-af (**B**), Fc4m-f (**C**), and FcAs-f (**D**). Each cell in the heatmap represents the mean duration between a pair of residues from different protein chains based on four independent molecular dynamics simulations (900 ns) for each complex. The CH3–CH3 interface is delineated by a green frame. The color bar indicates H-bond mean durations ranging from 0 to 900 ns. For visualization clarity, only interactions with a mean cumulative duration ≥ 45 ns are shown. Corresponding numerical values (mean ± SD) are provided in Supplementary Table S2.

**Figure 3:**
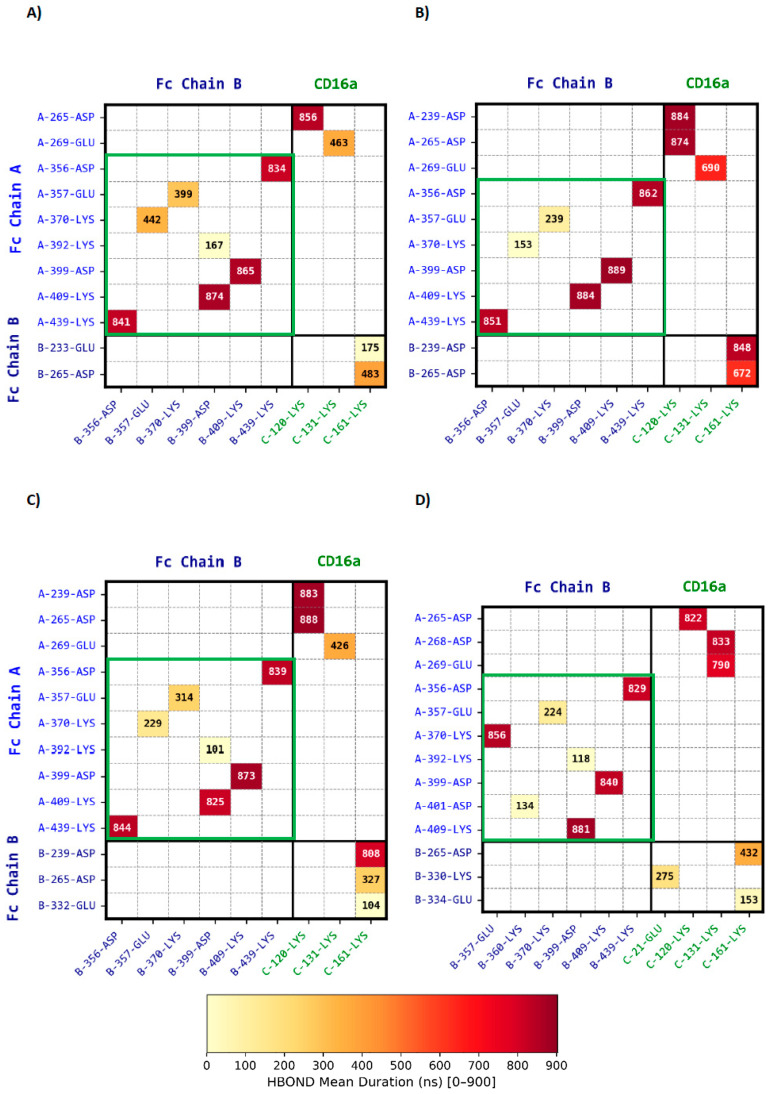
Heatmap representing intermolecular ionic interaction durations. The heatmap illustrates the average durations of ionic bond interactions at the inter-chain residue level over a 900 ns simulation over a 900 ns calculation within the Fc (Chains A & B) and CD16a on four distinct complexes: Fc-af (**A**), Fc4m-af (**B**), Fc4m-f (**C**), and FcAs-f (**D**). Each cell in the heatmap represents the mean duration between a pair of residues from different protein chains based on four independent molecular dynamics simulations (900 ns) for each complex. The color gradient from yellow to red indicates increasing interaction duration. The CH3–CH3 interface is delineated by a green frame. The color bar indicates ionic mean durations ranging from 0 to 900 ns. For visualization clarity, only interactions with a mean cumulative duration ≥ 45 ns are shown. Corresponding numerical values (mean ± SD) are provided in Supplementary Table S3.

**Figure 4:**
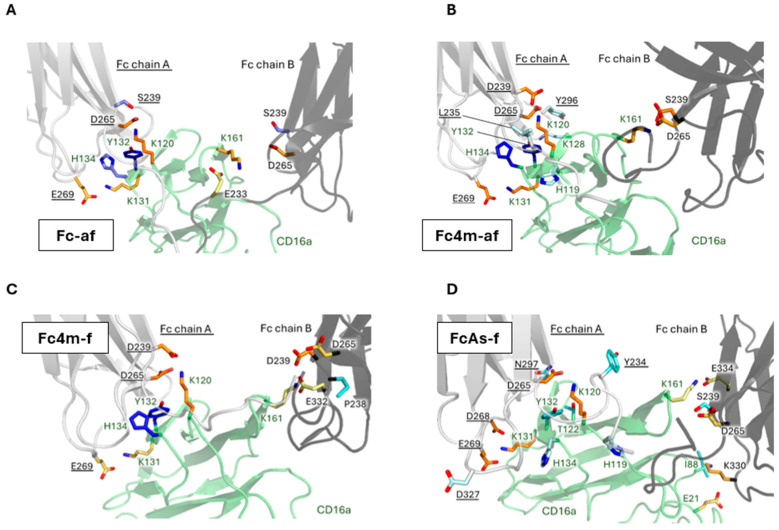
Amino Acid Interactions at the Fc–CD16a Interface. Residues involved in intermolecular interactions between Fc and CD16a are shown for each complex: Fc-af (**A**), Fc4m-af (**B**), Fc4m-f (**C**), and FcAs-f (**D**). Fc chain A is shown in light gray, Fc chain B in dark gray, and CD16a in green. Residues forming hydrogen bonds (blue) or ionic interactions (orange) are displayed when their cumulative interaction duration exceeds 200 ns during molecular dynamics simulations, highlighting the most persistent contacts at the Fc–CD16a interface. The last frame of the simulation is used to illustrate.

In this study, van der Waals (Figure S7A), hydrogen bonds (Figure 2A) and ionic interactions (Figure 3A) were analyzed for the afucosylated-wild-type Fc (Fc-af) over the final 900 ns of molecular dynamics (MD) simulations, applying a 10% occupancy cutoff using the RING program [46,47]. To further visualize interchain interactions, we scripted PyMOL3.7, to allow identification of key interaction hotspots (Figure 4A).

Within the Fc dimer, interchain hydrogen bonds stabilize the CH3-CH3 interface, reinforcing Fc structural integrity (Figure 2A, green zone and Figure S9A). Beyond this at the Fc-CD16a interface, transient hydrogen bonds between His268 and Tyr296 of Fc chain A and CD16a residues 127–132 suggest flexible, short-lived contacts (Figure 2A and Figure 4A). More stable interactions (300–600 ns) involve Gly236-Ser239 engaging Lys120 and His134 of CD16a. Among the most persistent, Asp265 forms a hydrogen bond with Tyr132, acting as a key stabilizing anchor. Similarly, on Fc chain B (Figure 2A), Leu235-Ser239 establish hydrogen bonds with Lys161, further strengthening receptor binding [26]. Moreover, the analysis of van der Waals interactions further supports the interaction pattern (Figure S7A) with Pro329 of Fc chain B tightly encaged by the proline sandwich Trp90 and Trp113 of CD16a, as previously reported [23,24].

Electrostatic interactions play a key role in Fc-CD16a stabilization. As shown on Figure 3A (green zone) and Figure S9A, the CH3-CH3 interface is reinforced by multiple salt bridges between Fc chains A and B, maintaining dimer integrity. At the receptor-binding site, Asp265 of Fc chain A forms a strong and persistent salt bridge with Lys120 (bleu zone). Additional medium-lived ionic interactions involve Glu269 and Lys131 of CD16a, enhancing electrostatic complementarity. Whereas Asp265 of Fc chain B interacts with Lys161 with moderate stability (orange zone). Further, Glu233 contributes to a short-lived contact with the receptor. Replicate means ± SD and full statistics with cross-complex Welch *t*-tests and unique-contact lists are reported in the Supplementary Information (Tables S3–S6).

Compared to prior structural studies and short MD simulations, our long-timescale trajectory provides a refined view of Fc-CD16a interactions, confirming key salt bridges while revealing that several predicted contacts (e.g., involving S239 on Fc chain B) are either absent or significantly less stable [38]. Binding in the afucosylated wild-type complex is dominated by a small set of persistent interactions, centered on Asp265 and lower-hinge residues, whereas most other contacts are transient and contribute only marginally to interface stabilization. This underscores the necessity of extended simulations to accurately resolve Fc-CD16a binding dynamics [27,33].

#### 3.2.2. Protein to Glycan Interactions

Structural biology and molecular dynamics studies have shown that Fc glycans modulate Fc conformation by maintaining its quaternary structure through the restriction of C_H_2 domain motional freedom via multiple intramolecular interaction networks. On Fc, the Asn297-linked glycan is a complex carbohydrate composed of a mannose (Man) and N-acetylglucosamine (GlcNAc) core, usually fucosylated. While conformational adjustments are possible for Fcγ receptor interactions, glycans primarily stabilize their respective Fc chain [33,39,48]. On CD16a, the glycan at N162 indirectly affects Fc binding affinity by constraining receptor flexibility [27].

Using our long-timescale simulations, we aimed to quantify protein–glycan contacts contributing to Fc stabilization and to assess how receptor and Fc glycans engage within the bound Fc–CD16a complexes. To this end, glycan modeling was restricted to PDB-resolved glycan structures [22,26] reflecting constrained glycan flexibility within the protein architecture. To achieve this, we systematically analyzed hydrogen bond interactions between glycans and protein chains across different Fc variants

Our Fc-af model includes an eight-sugar glycan at Asn297 on both Fc chains (A and B) and no fucose. For CD16a, we included a three-sugar glycan at N45 and an eight-sugar glycan, including core fucose, at N162. The durations of hydrogen bond interactions between protein residues of the Fc region and CD16a with glycan chains are presented in Figure S8. As well, glycan flexibility was quantified by computing the per-atom RMSF of the anomeric C1 for each monosaccharide across replicates (Figure S6).

As shown in Figure S8A for Fc-af, protein–glycan hydrogen bonds are predominantly intrachain: each Asn297 N-glycan engages mainly its own CH2 domain. The Fc chain-A glycan (CAR A) is strongly stabilized, with persistent contacts at the core (Asp265 ↔ GlcNAc1) and at the terminal arm (Glu258 ↔ Gal8), consistent with a locally locked conformation. By contrast, the chain-B glycan exhibits more transient contacts with C_H_2, indicating greater flexibility. CD16a glycans at N45 and N162 show limited intramolecular contact with a medium-lived interaction observed between Arg155 and GlcNAc2 on CAR C, and only short-lived intermolecular events are detected (e.g., Glu293 on Fc chain A with the second sugar of a CD16a glycan). *A stabilizing role of Arg155 interacting with the proximal GlcNAc residue of CAR C has been reported* [49], *although in our simulations this interaction involves GlcNAc2 rather than the innermost GlcNAc1 and remains dynamically labile within pre-formed complexes.* Consistent with this, per-atom RMSF of the anomeric C1 shows low dispersion for core sugars (GlcNAc1–2, Man3–4) and higher dispersion at termini, with CAR A Gal8 as a local outlier rather than evidence of global terminal convergence.

In relation with previous reports, no persistent hydrogen bonds involving Tyr296 of Fc chain A and B and a sugar moiety were detected in a dynamic context [24,26,27,39,50]. However, the distance between Tyr296 of Fc chain A and Man6 or Glc7 of CAR C from the CD16a glycan remains within 4–5 Å, confirming a spatial proximity, without hydrogen bond formation due to the orientation of Tyr296’s side chain. Nonetheless, as shown in Figure 2A, Tyr296 of Fc chain A engages Gly127 and Lys128 of CD16a. Thus, protein–glycan interactions in the afucosylated wild-type complex are predominantly intrachain and contribute to local stabilization of the CH2 domains rather than to direct Fc–CD16a binding.

#### 3.2.3. Glycan to Glycan Interactions

The glycan at Asn162 of CD16a is in immediate proximity, within interaction distance, to the glycan chain at Asn297 of Fc chain A, as previously reported [27]. No such spatial proximity is observed between the glycan on Fc chain B and the other glycans.

The direct intra- and inter-chain hydrogen bonds between glycans were quantified, distinguishing donor and acceptor atoms involved in these interactions. As illustrated in Figure S9, only a few carbohydrate-carbohydrate hydrogen bond interactions were detected, consistent with previous reports indicating that sugar-sugar interactions rarely play a dominant role in protein–protein complex formation [24,26,27]. Here, molecular dynamics simulations reveal additional transient glycan–glycan contacts that are absent in static crystallographic models. Notably, hydrogen bond interactions involving GlcNAc1 of CAR A with GlcNAc1 and 2 of CAR C were observed with moderate persistence (<300 ns), suggesting that while these contacts are not the primary stabilizing forces, they can transiently contribute to glycan positioning within the complex. Similarly, in crystal structures, the glycans of the two Fc chains exhibit minimal direct interactions. Overall, glycan–glycan contacts remain sparse and short-lived in the afucosylated wild-type complex.

### 3.3. Interactions Between the Afucosylated DFTE Fc Fragment and CD16a

#### 3.3.1. Protein to Protein Interactions

The Fc4m-af complex harbors the DFTE mutations on both Fc chains and lacks core fucosylation, resulting in an affinity in the nanomolar range for CD16a [11,29,44]. Among these mutations, only S239D and I332E may directly contribute to the central binding interface (Figure 1b). Throughout the remainder of the article, interactions are discussed only if they meet a significant difference by cross-complex Welch’s *t*-test (*p* < 0.05; see Supplementary Materials).

As shown on Figure 2 and Figure 3, panel B, the substitution of Ser239 by Asp establishes long-lived electrostatic interactions with Lys120 of CD16a for Fc chain A and Lys161 for Fc chain B, reinforcing the Fc-CD16a interface. In addition, Glu269 of Fc chain A exhibits a more prolonged interaction (~200 ns) with Lys131 of CD16a than in Fc-af, further enhancing electrostatic complementarity. All other contacts remain within statistical variability. On Fc chain A, only the Gly237 hydrogen bond is altered, becoming slightly weaker (Tables S4–S6). A direct visualization of these interactions is provided in Figure 4B.

Consistent with previous studies, the DFTE mutations reinforce electrostatic complementarity enhancing CD16a binding affinity, particularly through S239D [22,23,24,27]. However, our long-timescale simulations provide a precise characterization of this phenomenon, quantifying interaction persistence and enhancements. Notably, some interactions previously reported in short MD simulations, such as with I332E of Fc chain A, were absent in our extended 900 ns trajectories. Conversely, certain highly transient interactions that were observed within the first 100 ns but disappeared thereafter, underscoring the importance of long-timescale simulations to accurately capture the dynamic nature of Fc-CD16a binding.

Despite four mutations per Fc chain, only three of the eight substitutions actively contribute to new interactions, driving the observed affinity increase for this engineered Fc variant. Residue-level MM/GBSA analyses confirm that S239D mutation on both Fc chains provides the dominant enthalpic gains (toward Lys120 and Lys161) with no other residue showing a significant shift relative to Fc-af (Figure S12).

#### 3.3.2. Protein to Glycan and Glycan to Glycan Interactions

Over the 900-ns trajectories, Fc4m-af modifies protein to glycan hydrogen bonds in an asymmetric manner between the two Fc chains compared to the afucosylated-wild-type. On Fc chain A, the long-lived interaction between GlcNAc1 of CAR A and the key residue Asp265 is maintained, and contacts between the Gal8 antenna and residues in the 242–260 region remain comparable to those in Fc-af. In contrast, Fc chain B displays a clear increase in hydrogen bond stabilization with Gal8 of CAR B, involving hinge/CH2 residues, in particular the charged side chains of Lys246, Asp249, Glu258, Thr260 and Asp265, indicating a more anchored glycan conformation on this chain (Figure S8B). This stabilization of CAR B is consistent with the decreased RMSF of its terminal sugars (Figure S6). Additionally, glycan-CD16a self-interactions show slightly reduced lifetimes.

No significant alterations in glycan–glycan interactions were observed between the DFTE mutant and the afucosylated-wild-type complex (Figure S9B). The overall pattern of transient carbohydrate-carbohydrate contacts remains unchanged, with interactions involving GlcNAc1 of CAR A, GlcNAc1, and GlcNAc2 of CAR C displaying similar persistence times across the two complexes.

### 3.4. Interactions Between the Fucosylated DFTE Fc Fragment and CD16a

#### 3.4.1. Protein to Protein Interactions

Core fucosylation of the Asn297-linked Fc glycan significantly impairs CD16a binding and effector function [11,28,51]. The removal of core fucose has been shown to enhance protein binding, leading to increased ADCC potency [24,26]. Structural and molecular dynamics studies suggest that fucosylation primarily influences CD16a engagement by modulating protein–protein and protein–glycan interactions, particularly through conformational changes in the receptor-associated glycan at Asn162 [24,27,33,52].

In the Fc4m-f complex, which carries the DFTE mutations and includes core fucosylation on both Fc chains, our long-timescale MD simulations reveal distinct alterations in the Fc-CD16a interaction network. Non-covalent CH3-CH3 interactions remain largely unaffected, indicating preserved Fc dimer integrity (Figure 2 and Figure 3, panel C). Visualization of interactions is shown on Figure S10C.

At the Fc-CD16a interface, the S239D mutation continues to promote long-lived contacts with Lys120 of CD16a for Fc chain A and Lys161 for Fc chain B, maintaining the central reinforcement observed in the non-fucosylated mutated Fc4m-af complex. In contrast, peripheral contacts are selectively weakened. Glu269 of Fc chain A shows reduced interaction persistence with Lys131 of CD16a, and Asp265 of Fc chain B displays diminished stability in its interaction with Lys161. At the hydrogen bond level, a rapid interaction loss is noted between Tyr296 of Fc chain A and Lys128 of CD16a (Figure 2C and Figure 3C). On Fc chain B, residues 235–240 show a redistribution of contacts with Lys161 of CD16a, with long-lived hydrogen bonds seen in Fc4m-af being replaced by a set of more transient interactions. Interestingly, van der Waals interactions (Figure S7C) involving Pro329 of Fc chain B show a marked decrease, particularly within the proline sandwich formed by Trp90 and Trp113 of CD16a, indicating a weakened hydrophobic contribution to Fc-CD16a binding. Residue-level MM/GBSA analyses indicate that the enthalpic contributions from S239D on both chains remain essentially unchanged upon fucosylation, whereas Glu269, and Tyr296 of Fc A and Asp265 of Fc B display only minor variations not statistically significant between Fc4m-af and Fc4m-f (Figure S12). Direct visualization of interactions is represented in Figure 4C.

In conclusion, while the central interface around Lys120 and Lys161 is largely preserved, fucosylation attenuates peripheral stabilizing interactions. These include weakened hydrophobic packing near Pro329 of Fc chain B and reduced contributions involving Tyr296 and Glu269 of Fc chain A, in agreement with previous structural studies [22,24,27,33,52].

#### 3.4.2. Protein to Glycan and Glycan to Glycan Interactions

Fucosylation of Fc N-glycans is known to reduce CD16a binding affinity and, consequently, impair ADCC activity [35,52]. Sakae et al. proposed that this loss of function arises from increased flexibility of the CD16a glycan at Asn162 and positioning further from the Fc glycan, thus disrupting optimal intermolecular carbohydrate-carbohydrate interactions.

In the fucosylated DFTE complex Fc4m-f, comparative heatmaps of protein–to–glycan hydrogen bonds (Figure S8, panels B and C) indicate that the long-lived anchoring between Asp265 and GlcNAc1 of CAR A is moderately weakened but remains present for most of the trajectory. Fc chain A also exhibits a loss of two hydrogen bonds with GlcNAc5 of the CD16a CAR C glycan, highlighting a disruption of stabilizing contacts at the receptor glycan. Fc chain B displays a slight reinforcement and distribution of interactions between GlcNAc 1–2 with Asp265 and Arg301 of CD16a, consistent with the presence of fucose at the beginning of this glycan arm. Further modifications are observed in the interactions involving the CD16a glycan at Asn162 with the loss of the Arg155–GlcNAc2 (CAR C) hydrogen bond. Interestingly, the C1-atom RMSF profiles (Figure S6) show slightly reduced fluctuations for CAR B and increased flexibility for the distal residues of CAR C in Fc4m-f.

Marked alterations in glycan–glycan interactions are observed upon the presence of core fucose (Figure S9, panels B and C). The most significant impact is the loss of key intermolecular hydrogen bonds, specifically between GlcNAc1 and GlcNAc2 of the Fc glycan (CAR A) and GlcNAc4 of the CD16a CAR C glycan, and between GlcNAc1 of the Fc CAR A glycan and GlcNAc1–2 of the CD16a CAR C glycan. The presence of the fucose on the Fc chain A glycan likely prevents these interactions either sterically or by increasing the glycan’s spatial separation. Additionally, a rearrangement of intramolecular hydrogen bonds within Fc chain A and B glycans was observed.

These results confirm and refine the notion that Fc core fucosylation imposes steric constraints that alter glycan-mediated stabilization of the Fc–CD16a interface, disrupting key hydrogen bond networks. The loss of intermolecular glycan–glycan interactions, together with reduced glycan–protein contacts, illustrates how fucosylation may contribute to weakening receptor engagement through a complex redistribution of stabilizing interactions. This is consistent with previous studies describing increased flexibility of the CD16a Asn162 glycan, further supporting the idea that fucosylation remodels the local glycan environment in a way that reduces optimal receptor binding [24,27,52].

### 3.5. Interactions Between the Asymmetric Fc Fragment and CD16a

#### 3.5.1. Protein to Protein Interactions

Therapeutic antibodies such as rituximab rely on the optimal ratio of binding to activating FcγRs (e.g., FcγRIIIa/CD16a) versus inhibitory FcγRs (FcγRIIb/CD32b) to achieve maximal antitumor efficacy. However, symmetric Fc mutations such as S239D and I332E, while enhancing FcγRIIIa binding, also increase affinity for FcγRIIb, potentially reducing therapeutic efficiency [35,36]. To address this limitation, Mimoto et al. engineered an asymmetric Fc that demonstrated improved affinity for both FcγRIIIa allotypes (V/F158) and superior or comparable ADCC without increasing FcγRIIb affinity.

Our simulations with the asymmetric FcAs-f complex aim to elucidate how these mutations influence the binding interface with CD16a. This model includes core fucosylation on the glycan sites of both Fc chains and features eight substitutions on Fc chain A, most notably S239M, and nine distinct mutations on Fc chain B, including K334E. We compared the durations of hydrogen bond, ionic interactions, and van der Waals interactions with those in the symmetric Fc4m-f complex over the course of the molecular dynamics simulations.

At the CH3-CH3 dimer interface, as in Fc4m-f, ionic interactions, particularly salt bridges involving Lys409 and Asp399, are well maintained (Figure 2, panels C and D and Figure S10, panels A and D). A slight decrease in hydrogen bond occupancy is observed in the asymmetric Fc, notably near Tyr407 of Fc chain B. However, this reduction is offset by the presence of an additional disulfide bond between Cys349 of Fc chain A and Cys356 of Fc chain B, which contributes to dimer stability.

When compared to Fc4m-f, FcAs-f displays a redistribution of the hydrogen bond networks (Figure 2D). Moreover, the hydrogen bond between Asp265 of Fc chain A and Tyr132 of CD16a is reduced by nearly 400 ns. Also, Fc chain B of FcAs-f establishes a broader contact region extending from Leu235 to Val240, though with shorter interaction durations, with Lys161.

In FcAs-f, position 239 is mutated to methionine on Fc chain A and remains a serine on Fc chain B, resulting in the loss of the two central electrostatic contacts with CD16a observed in Fc4m-af and Fc4m-f. However, the key interaction between Asp265 of Fc chain A and Lys120 of CD16a remains strongly populated. Additionally, in peripheral regions of the interface, the H268D mutation on Fc chain A introduces a new strong ionic interaction with Lys131 of CD16a and reinforces pre-existing interactions mediated by Glu269. Finally, the A330K mutation on Fc chain B establishes a novel short-lived ionic contact with Glu21 of CD16a (Figure 4D).

Despite reductions in hydrogen bonding and ionic interactions within the central contact region, the asymmetric Fc retains high nanomolar affinity [35,36]. To investigate potential compensatory mechanisms, van der Waals interactions were analyzed. In the FcAs-f complex, these contacts are redistributed across a patch of mutated hydrophobic residues within Fc chain A (residues 234–236), particularly involving the mutated Trp236. However, long-range interactions with His134 of CD16a are either lost or markedly reduced (Figure S7D). The local hydrophobic patch around Trp236 is illustrated in Figure S11, which displays the persistent hydrophobic interactions (>200 ns cumulative duration) between Fc chain A and CD16a over the course of the MD simulations. MM/GBSA further shows that these hydrophobic rearrangements yield no significant enthalpic gains, with only the H268D residue of Fc chain A forming a detectable stabilizing interaction with Lys131 of CD16a.

Consequently, the preservation of high affinity in the FcAs-f complex likely originates from peripheral contributions, notably involving the 268–269 region of Fc chain A and the hydrophobic cluster around Trp236 on Fc chain A which provides localized stabilization. However, it should be noted that the observations from this long-timescale molecular dynamics study differ markedly from the conclusions of the crystallographic study by Mimoto et al., particularly regarding the involvement of S239M on Fc chain A and D270E/K326D on Fc chain B [35,36]. Also, no stable interaction involving K334E was detected, suggesting a minimal direct contribution to CD16a engagement in this context. Despite the presence of multiple polar or charged residues theoretically capable of engaging in hydrogen bonding or electrostatic interactions, only a limited subset of mutations in the asymmetric FcAs-f model establish persistent direct contacts with CD16a under dynamic conditions.

#### 3.5.2. Protein to Glycan and Glycan to Glycan Interactions

When compared with Fc4m-f, which also carries a core fucose on each Fc chain, subtle yet specific differences emerge in the glycan hydrogen-bond network of the FcAs-f complex (Figure S8, panels C and D). On Fc chains A and B, the N-glycans linked to Asn297 predominantly form intrachain hydrogen bonds, indicating a preserved structural role in maintaining Fc conformation. However, a slight reduction in the number of short-lived hydrogen bonds involving Gal8 is observed relative to Fc4m-f, suggesting a marginal loosening of glycan anchoring. In addition, Fc chain B displays two extra transient hydrogen bonds involving GlcNAc5 and Man6 of the CAR B glycan, indicating a local compensation through short-range stabilization. On the CD16a side, CAR C no longer engages protein–glycan hydrogen bonds above our 90-ns cutoff in FcAs-f.

The hydrogen-bond profiles between carbohydrate chains in FcAs-f are further reduced compared with Fc4m-f, with only three very short-lived glycan–glycan contacts detected (Figure S9). Consistently, the C1-atom RMSF profiles of the four core glycans (Figure S6) show only modest differences between Fc4m-f and FcAs-f: the terminal residues of CAR B display slightly decreased fluctuations in FcAs-f, whereas CAR C remains more flexible than in the afucosylated complexes.

Taken together, these observations indicate that the FcAs-f complex does not exhibit enhanced glycan-mediated stabilization—neither through intrachain anchoring, receptor-glycan contacts, nor inter-glycan hydrogen bonds. The observed high-affinity binding of FcAs-f is therefore most likely driven by alternative structural mechanisms, primarily rooted in selective protein–protein interactions.

## 4. Discussion

We used the afucosylated-wild-type Fc-CD16a complex (Fc-af) as a reference with baseline affinity in the low nanomolar range (~70–100 nM) [24,29,35]. Previous structural studies have described an asymmetric interface, organized around two conserved electrostatic anchors: Asp265 of Fc engaging Lys120 and Lys161 of CD16a. In addition, Trp90 of CD16a inserts into a hydrophobic pocket formed by Leu235 and Pro329 of Fc chain A, thereby stabilizing the Leu234-Pro238 segment [23,25,26]. Here, μs-scale MD uncovers and measures a persistent asymmetry in contact networks and lifetimes, yielding a dynamic atlas that refines prior models.

On the Fc-af complex, Fc chain A sustains a dense and durable network of interactions, particularly in the central zone where Asp265 forms stable salt bridges with Lys120 (Figure 2, Figure 3 and Figure S7). Notably, we identified a persistent hydrogen bond cluster upstream of Ser239 on chain A (residues 236–238), consistent with prior structural observations describing the stabilization of this segment within the complex [23,25]. Peripheral zones, including residues such as Glu269 interacting with Lys131 of CD16a, Tyr296 and Pro329 of Fc contribute to local alignment through a combination of electrostatic interactions and hydrophobic complementarity. This observation is consistent with previous reports showing Pro329 stably embedded in the Trp90/Trp113 cleft of CD16a. Tyr296 of Fc chain A forms only short-lived hydrogen bonds with Lys128 and Gly127 of CD16a, in agreement with its known conformational flexibility [22,24,27]. By contrast, Fc chain B contributes shorter-lived or intermittent contacts, principally via residues engaging Lys161 of CD16a.

Glycan interactions were found to be primarily intrachain, with each glycan stabilizing its own carrier chain. The duration-based analysis reveals that the glycan linked to Asn297 of Fc chain A forms two long-lived anchoring points: GlcNAc1 engages Asp265 and Gal8 interacts with several residues, contributing to local stabilization. In contrast, the glycan on chain B is more mobile and displays weaker anchoring (Figures S8 and S9). The glycan linked to Asn162 of CD16a shows only a single stable contact via GlcNAc2. Last, our study shows that short-range inter-glycan contacts do occur, particularly between the glycan linked to Asn297 on Fc chain A and the glycan on CD16a (Asn162). However, their lifetimes remain below 260 ns (Figure S9), indicating that they are transient and not structurally stabilizing. Consistent with these findings, recent MD-based studies have similarly concluded that glycan–glycan interactions do not function as dominant stabilizing elements of the Fc-CD16a complex [27,33]. These findings support the view that the main contribution of glycans to affinity is mediated through their influence on local flexibility and steric organization, rather than through sustained interchain carbohydrate bridges.

Taken together, Fc-af presents an interaction topology based on a cohesive central zone, flexible peripheral contacts, and glycan mobility that primarily stabilizes its carrier chain. These results support and extend prior structural interpretations by offering a dynamic quantification of interaction persistence. This baseline framework enables us to dissect how specific Fc mutations and glycan modifications alter this dynamic architecture in subsequent engineered complexes. Importantly, our simulations focus on the dynamic stability of pre-formed complexes, providing insight into the persistence of inter-residue contacts in the bound state rather than the kinetics of complex formation. Consequently, the wild-type Fc with core fucosylation, which binds CD16a with only micromolar affinity, was not included in this analysis as its transient binding would not allow for meaningful evaluation of interaction dynamics.

In contrast to Fc-af, the Fc4m-af complex incorporates the S239D, H268F, S324T, and I332E mutations on both Fc chains in an afucosylated background with nanomolar binding affinity to CD16a (1–3 nM). On Fc chain A, the electrostatic core is reinforced at two conserved anchors, S239D establishes a persistent salt bridge with Lys120 of CD16a, while Asp265 maintains a stable interaction with Lys161 of CD16a [22] (Figure 2, Figure 3 and Figure S7). A specific feature is the stabilization of a hydrogen bond involving residue Gly236 on chain A. The peripheral electrostatic interaction between Glu269, adjacent to the H268F, and Lys131 of CD16a is strongly stabilized, suggesting a local gain over the original histidine residue. On chain B, two principal ionic contacts exist, one being reinforced by S239D. These interactions clearly contribute to the central interface, but the asymmetric engagement of the two Fc chains is still present. Finally, contrary to suggestions from crystallography or short-timescale MD, I332E of Fc does not exhibit a marked or persistent direct interaction with CD16a in our trajectories. This result should be interpreted within the scope of the present analysis, which focuses on the persistence and redistribution of inter-residue interactions in pre-formed Fc–CD16a complexes. Accordingly, the absence of persistent contacts involving I332E constrains its role in the stabilized bound state, without addressing possible contributions at earlier, transient stages of the interaction. In terms of glycan dynamics, the Asn297-linked glycans on both Fc chains show improved anchoring within their respective chains, especially on chain B, via GlcNAc1 and Gal8 (Figures S8 and S9). Although these contacts do not persist beyond a few hundred nanoseconds, they may contribute transiently to interface alignment.

These results highlight that DFTE mutations increase affinity primarily by enhancing the duration and density of key electrostatic and hydrogen interactions at the interface, in line with prior short-timescale and structural observations. However, our μs-scale analysis quantifies interaction lifetimes for each Fc chain at the pair level and aggregates evidence across independent replicas, thereby resolving the persistent A/B asymmetry in Fc engagement with CD16a, both at the central interface and in peripheral motifs, and providing a chain-resolved account of how these mutations enhance complex stability.

While the same DFTE mutations are present as in Fc4m-af, the Fc4m-f complex displays a reduced binding stability to CD16a, consistent with the well-documented inhibitory effect of core fucosylation [2,24,27]. Although no direct affinity values are available for the fucosylated DFTE variant (Fc4m-f), previous studies have shown that core fucosylation reduces Fc-CD16a binding by up to tenfold in wild-type IgG1 Fc contexts. At the protein–protein interface, our study reveals that ionic interactions such as S239D-Lys120 and Asp265-Lys120 on chain A, and Asp239-Lys161 on chain B, remain highly persistent (Figure 2, Figure 3 and Figure S7). However, the Glu269-Lys131 contact on chain A is significantly reduced in duration, the hydrogen bond involving Gly236 is moderately shortened, and the ionic interaction between Asp265 on chain B and Lys161 of CD16a shows decreased persistence. These changes suggest a local destabilization of the peripheral interaction network, though the central binding core remains intact. Moreover, van der Waals contacts deteriorate more markedly. Pro329 of Fc chain B loses stable interaction with the hydrophobic cleft formed by Trp90 and Trp113 of CD16a, indicating a breakdown of peripheral hydrophobic anchoring compared to the afucosylated variant. Regarding glycan interactions, fucosylation alters intrachain and interchain engagement of Fc chain A (Figures S8 and S9), particularly those involving GlcNAc5 of the CD16a Car C glycan. Moreover, glycan–glycan hydrogen bonds involving Car A and Car C are no longer detected, consistent with the spatial disruption introduced by the core fucose. Together, these effects support the view that core fucosylation impairs Fc-CD16a binding by restricting the formation and persistence of auxiliary glycan-mediated contacts, both within and across chains, while the central electrostatic interactions remain largely preserved.

Taken together, Fc4m-f illustrates how core fucosylation overrides part of the DFTE-induced stabilization by perturbing peripheral electrostatic, hydrophobic, and glycan-mediated interactions. This underscores the importance of considering affinity-enhancing mutations and glycoengineering as complementary strategies when optimizing Fc-receptor interactions.

The FcAs-f complex offers a compelling contrast to the previous variants. Despite the absence of the central S239D mutation, and despite the presence of core fucose on Fc, the complex maintains high-affinity binding in the nanomolar range [35,36]. This result challenges the notion that enhanced affinity necessarily requires either strong central electrostatic anchoring or removal of fucose, and suggests an alternative mode of interface optimization based on asymmetry and spatial reorganization.

At the protein–protein interface, the S239 position on chain A is mutated to methionine in FcAs-f. At the central interface, the electrostatic interactions resemble those observed in the afucosylated-wild-type Fc-af complex, with only Asp265 of chain A maintaining strong binding to Lys120 of CD16a (Figure 2, Figure 3 and Figure S7). Moreover, hydrogen bonds at the central interface are less persistent. However, a novel hydrophobic cluster forms around mutated Trp236 on Fc chain A, contributing to the stabilization of the central contact zone. This hydrophobic patch, absent in both the afucosylated-wild-type and DFTE complexes, may act as a conformational anchor (Figure S11). Peripheral electrostatic contributions are also restructured. On chain A, the H268D substitution enables a strong and persistent interaction with Lys131 of CD16a, reinforcing the Glu269 region, which was partially destabilized by fucosylation in the Fc4m-f complex. A secondary electrostatic contact, established by A330K on chain B with Glu21 of CD16a, further contributes to interface stabilization, although it remains short-lived. Together, these peripheral interactions suggest a redistribution of stabilizing forces across a broader interface. From a glycan perspective, the presence of core fucose in FcAs-f, as with Fc4m-f only low local stabilization is observed for CAR B, whereas CAR C is more flexible than in the afucosylated complexes (Figure S6).

Altogether, these observations suggest that in the FcAs-f complex, high-affinity binding arises from a reorganization of stabilizing forces across an asymmetrical interface, combining localized hydrophobic reinforcement and peripheral electrostatic compensation, despite the presence of core fucose and the absence of the canonical central S239D anchor. While Mimoto et al. [35] demonstrated the structural feasibility of such asymmetry, our dynamic simulations confirm and extend this by quantifying the persistence of these interactions and their spatial redistribution in solution.

## 5. Conclusions

Our μs-timescale molecular dynamics establish an atomistic dynamic atlas of Fc–CD16a recognition, delineating stabilizing contacts and quantifying how DFTE engineering, core fucosylation, and asymmetric designs rewire these networks. This study extends prior sub-microsecond MD studies focused on single Fc variants with a chain-resolved, multi-variant dynamic atlas of Fc–CD16a recognition. Across the DFTE dimer, only three substitutions make persistent, chain-resolved contributions, with S239D on both Fc chains creating two major electrostatic anchors and H268F on chain A indirectly strengthening the peripheral Glu269 to Lys131 contact. In the asymmetric FcAs-f, loss of central electrostatics is compensated by a localized hydrophobic cluster centered on Trp236 on chain A together with reinforced peripheral electrostatics around H268D and Glu269. Consistent with prior structural and MD work showing that Fc N-glycans act chiefly as intrachain stabilizers, our duration-based analysis resolves chain specificity and shows that DFTE tightens the Asn297 glycan on chain B, whereas core fucosylation weakens auxiliary protein–glycan contacts and abolishes the few transient glycan–glycan bridges observed in afucosylated contexts. In this study, interfacial waters were not analyzed, despite evidence of stabilizing water bridges at Fc–receptor interfaces [53]. Mapping water occupancy, lifetimes, and water-bridged networks at Fc–CD16a is a pertinent extension to complete the atlas.

Importantly, the interaction durations reported here are not intended as surrogates for binding free energy, but as dynamic descriptors of contact stability within pre-formed complexes; energetic contributions were instead assessed separately using residue-level MM/GBSA analyses, which explicitly account for electrostatic, van der Waals, and solvation effects.

Altogether, this chain-resolved framework and multi-variant dynamic atlas of Fc–CD16a recognition support rational Fc engineering. With central anchors S239D and Asp265, near-optimal, tunable peripheries emerge, including an electrostatic rim on chain A at positions 268–269 rewired toward Lys131, a hydrophobic slot on chain A at positions 234–236, and an A330K sector to introduce a new interaction. This outlines possible routes to reinforce FcγRIIIa affinity while potentially modulating FcγRIIb engagement [30,36] and to guide Fc backbones for ex vivo NK-arming protocols [29].

## Data Availability

Analysis scripts (Python/RING/PyMOL) are custom in-house tools and are not publicly distributed. All processed data underlying the figures and tables are provided in the Supplementary Information, and additional derived data can be shared upon reasonable request.

## Supplementary Materials

The following supporting information can be downloaded at: https://www.mdpi.com/article/10.3390/antib15010017/s1, **Supplementary File S1:** Microsecond Dynamics of Fc–CD16a Recognition: Impact of Mutations, Core Fucosylation, and Fc Asymmetry. This file contains detailed computational procedures (model building, MD simulation setup, and trajectory analyses), protein sequences/mutations and glycan compositions, an overview of simulated systems, extended stability/convergence analyses, and complete interaction-network/statistical analyses (Supplementary Tables S1–S9 and Supplementary Figures S1–S12). **Table S1**. Overview of the MD systems (composition and key simulation parameters for Fc-af, Fc4m-af, Fc4m-f, and FcAs-f; system size, numbers of atoms/residues for each component, frames, and sampling). **Figure S1**. Cα RMSD of Fc-af, Fc4m-af, Fc4m-f, and FcAs-f complexes during the NPT production run (900 ns; 100 ns excluded) for four replicas (D1–D4). **Figure S2**. Cα RMSF for Fc chain A, Fc chain B, and CD16a for each complex (Fc-af, Fc4m-af, Fc4m-f, FcAs-f) across four replicas (900 ns; 100 ns excluded). **Figure S3**. Radius of gyration (Rg) profiles of the four Fc–CD16a complexes over the 900 ns NPT production run (four replicas per complex). **Figure S4a**. Average Cα RMSD for Fc chain A, Fc chain B, and CD16a (mean over four 900 ns replicas) for each complex. **Figure S4b**. Average Cα RMSF profiles (mean over four 900 ns replicas) for Fc chain A, Fc chain B, and CD16a; mutation sites indicated for Fc4m-af and Fc4m-f. **Figure S4c**. Average radius of gyration (Rg) for Fc-af, Fc4m-af, Fc4m-f, and FcAs-f (mean over four 900 ns replicas). **Figure S5a**. Solvent accessible surface area (SASA) of the full protein complexes (including glycans) over 900 ns for four replicas (D1–D4). **Figure S5b**. SASA profiles for individual glycan chains (CAR A, CAR B, CAR C, CAR D) over 900 ns for four replicas (D1–D4) and for each complex. **Figure S6**. RMSF (Å) of the anomeric C1 atom of each monosaccharide for CAR A–D over four independent 900 ns replicas (D1–D4) for each Fc glycovariant (Fc-af, Fc4m-af, Fc4m-f, FcAs-f). **Table S2**. Intermolecular protein–protein hydrogen bonds between different chains (mean duration ± SD over four replicas; interactions retained with a 90 ns cutoff over the 900 ns analysis window). **Table S3**. Intermolecular protein–protein ionic interactions (salt bridges) between different chains (mean duration ± SD over four replicas; 90 ns cutoff over 900 ns). **Table S4-1**. Cross-complex Welch’s *t*-tests on protein–protein hydrogen bond durations (two-sided; unequal variances; only significant comparisons, *p* < 0.05). **Table S4-2**. Unique protein–protein hydrogen bonds (pairs present in exactly one complex; mean ± SD over four replicas). **Table S5-1**. Cross-complex Welch’s *t*-tests on protein–protein ionic interaction durations (two-sided; unequal variances; only significant comparisons, *p* < 0.05). **Table S5-2**. Unique protein–protein ionic interactions (pairs present in exactly one complex; mean ± SD over four replicas). **Figure S7**. Heatmap of intermolecular protein–protein van der Waals interaction durations (mean ± SD over four replicas; 180 ns visualization cutoff) for Fc-af (A), Fc4m-af (B), Fc4m-f (C), and FcAs-f (D). **Table S6-1**. Cross-complex Welch’s *t*-tests on protein–protein van der Waals interaction durations (two-sided; unequal variances; only significant comparisons, *p* < 0.05). **Table S6-2**. Unique protein–protein van der Waals interactions (pairs present in exactly one complex; mean ± SD over four replicas). **Figure S8**. Heatmap of hydrogen bond interaction durations between protein residues (Fc chains A/B; CD16a chain C) and glycan sugars (mean ± SD over four replicas; interactions shown above the 90 ns cutoff) for Fc-af (A), Fc4m-af (B), Fc4m-f (C), and FcAs-f (D). **Table S8-1**. Cross-complex Welch’s *t*-tests on protein–glycan hydrogen bond durations (two-sided; unequal variances; only significant comparisons, *p* < 0.05). **Table S8-2**. Unique protein–glycan hydrogen bonds (pairs present in exactly one complex; mean ± SD over four replicas). **Figure S9**. Heatmap of hydrogen bond interaction durations between glycan sugars (intra- and inter-glycan) for Fc-af (A), Fc4m-af (B), Fc4m-f (C), and FcAs-f (D) (mean ± SD over four replicas; interactions shown above the 90 ns cutoff). **Table S9-1**. Cross-complex Welch’s *t*-tests on glycan–glycan hydrogen bond durations (two-sided; unequal variances; only significant comparisons, *p* < 0.05). **Table S9-2**. Unique glycan–glycan hydrogen bonds (pairs present in exactly one complex; mean ± SD over four replicas). **Figure S10**. Persistent intermolecular interactions between Fc chains A and B at the CH3–CH3 interface (hydrogen bonds in blue; ionic interactions in orange) shown for each complex; interactions retained with cumulative duration > 200 ns over the MD simulations. **Figure S11**. Persistent hydrophobic interactions at the FcA–CD16a interface in the FcAs-f complex, centered on Trp236; hydrophobic contacts retained with cumulative duration > 200 ns. **Figure S12**. Pairwise MM/GBSA residue interaction enthalpies (ΔH, kcal·mol^−1^) between Fc residues (chains A/B) and CD16a residues computed over 100–1000 ns (91 snapshots at 10 ns intervals); generalized Born model with residue-pair decomposition; entropic terms not included; only interactions with |ΔH| ≥ 0.1 kcal·mol^−1^ displayed.

## Abbreviations

DFTE: Mutations of Fc on chains A and B, S239D, H268F, S324T, I332E. Man (Mannose), GlcNAc (N-Acetylglucosamine), Gal (Galactose), Fuc (Fucose), Glc (Glucose), Neu5Ac (N-Acetylneuraminic acid), Fc (Fragment crystallizable), Fab (Fragment antigen-binding), FcγR (Fc gamma receptor), ADCC (Antibody-dependent cellular cytotoxicity), IgG (immunoglobulin G), human IgG (hIgG). MD (Molecular Dynamics), PDB (Protein Data Bank), RMSD (Root Mean Square Deviation), RMSF (Root Mean Square Fluctuation).

## References

[1] Edelman G.M. (1971). ANTIBODY STRUCTURE AND MOLECULAR IMMUNOLOGY*. Ann. N. Y. Acad. Sci..

[2] Desjarlais J.R., Lazar G.A. (2011). Modulation of Antibody Effector Function. Exp. Cell Res..

[3] Schroeder H.W., Cavacini L. (2010). Structure and Function of Immunoglobulins. J. Allergy Clin. Immunol..

[4] Wagner E.K., Maynard J.A. (2018). Engineering Therapeutic Antibodies to Combat Infectious Diseases. Curr. Opin. Chem. Eng..

[5] Bruhns P., Jönsson F. (2015). Mouse and Human FcR Effector Functions. Immunol. Rev..

[6] Nimmerjahn F., Ravetch J.V. (2008). Fcγ Receptors as Regulators of Immune Responses. Nat. Rev. Immunol..

[7] Diebolder C.A., Beurskens F.J., De Jong R.N., Koning R.I., Strumane K., Lindorfer M.A., Voorhorst M., Ugurlar D., Rosati S., Heck A.J.R. (2014). Complement Is Activated by IgG Hexamers Assembled at the Cell Surface. Science.

[8] Liu R., Oldham R., Teal E., Beers S., Cragg M. (2020). Fc-Engineering for Modulated Effector Functions—Improving Antibodies for Cancer Treatment. Antibodies.

[9] Patel K.R., Roberts J.T., Barb A.W. (2019). Multiple Variables at the Leukocyte Cell Surface Impact Fc γ Receptor-Dependent Mechanisms. Front. Immunol..

[10] Alemán O.R., Mora N., Rosales C. (2021). The Antibody Receptor Fc Gamma Receptor IIIb Induces Calcium Entry via Transient Receptor Potential Melastatin 2 in Human Neutrophils. Front. Immunol..

[11] Shields R.L., Namenuk A.K., Hong K., Meng Y.G., Rae J., Briggs J., Xie D., Lai J., Stadlen A., Li B. (2001). High Resolution Mapping of the Binding Site on Human IgG1 for FcγRI, FcγRII, FcγRIII, and FcRn and Design of IgG1 Variants with Improved Binding to the FcγR. J. Biol. Chem..

[12] Mellor J.D., Brown M.P., Irving H.R., Zalcberg J.R., Dobrovic A. (2013). A Critical Review of the Role of Fc Gamma Receptor Polymorphisms in the Response to Monoclonal Antibodies in Cancer. J. Hematol. Oncol..

[13] Orange J.S. (2008). Formation and Function of the Lytic NK-Cell Immunological Synapse. Nat. Rev. Immunol..

[14] Zhu H., Blum R.H., Bjordahl R., Gaidarova S., Rogers P., Lee T.T., Abujarour R., Bonello G.B., Wu J., Tsai P.-F. (2020). Pluripotent Stem Cell–Derived NK Cells with High-Affinity Noncleavable CD16a Mediate Improved Antitumor Activity. Blood.

[15] Cerny T., Borisch B., Introna M., Johnson P., Rose A.L. (2002). Mechanism of Action of Rituximab. Anticancer Drugs.

[16] Musolino A., Naldi N., Bortesi B., Pezzuolo D., Capelletti M., Missale G., Laccabue D., Zerbini A., Camisa R., Bisagni G. (2008). Immunoglobulin G Fragment C Receptor Polymorphisms and Clinical Efficacy of Trastuzumab-Based Therapy in Patients With HER-2/*Neu*–Positive Metastatic Breast Cancer. J. Clin. Oncol..

[17] López-Albaitero A., Lee S.C., Morgan S., Grandis J.R., Gooding W.E., Ferrone S., Ferris R.L. (2009). Role of Polymorphic Fc Gamma Receptor IIIa and EGFR Expression Level in Cetuximab Mediated, NK Cell Dependent in Vitro Cytotoxicity of Head and Neck Squamous Cell Carcinoma Cells. Cancer Immunol. Immunother..

[18] Taylor R.J., Chan S.-L., Wood A., Voskens C.J., Wolf J.S., Lin W., Chapoval A., Schulze D.H., Tian G., Strome S.E. (2009). FcγRIIIa Polymorphisms and Cetuximab Induced Cytotoxicity in Squamous Cell Carcinoma of the Head and Neck. Cancer Immunol. Immunother..

[19] Moore G.L., Chen H., Karki S., Lazar G.A. (2010). Engineered Fc Variant Antibodies with Enhanced Ability to Recruit Complement and Mediate Effector Functions. mAbs.

[20] Wang X., Mathieu M., Brezski R.J. (2018). IgG Fc Engineering to Modulate Antibody Effector Functions. Protein Cell.

[21] Chen D., Zhao Y., Li M., Shang H., Li N., Li F., Wang W., Wang Y., Jin R., Liu S. (2021). A General Fc Engineering Platform for the next Generation of Antibody Therapeutics. Theranostics.

[22] Ahmed A.A., Keremane S.R., Vielmetter J., Bjorkman P.J. (2016). Structural Characterization of GASDALIE Fc Bound to the Activating Fc Receptor FcγRIIIa. J. Struct. Biol..

[23] Sondermann P., Huber R., Oosthuizen V., Jacob U. (2000). The 3.2-Å Crystal Structure of the Human IgG1 Fc Fragment–FcγRIII Complex. Nature.

[24] Ferrara C., Grau S., Jäger C., Sondermann P., Brünker P., Waldhauer I., Hennig M., Ruf A., Rufer A.C., Stihle M. (2011). Unique Carbohydrate–Carbohydrate Interactions Are Required for High Affinity Binding between FcγRIII and Antibodies Lacking Core Fucose. Proc. Natl. Acad. Sci. USA.

[25] Radaev S., Motyka S., Fridman W.-H., Sautes-Fridman C., Sun P.D. (2001). The Structure of a Human Type III Fcγ Receptor in Complex with Fc. J. Biol. Chem..

[26] Mizushima T., Yagi H., Takemoto E., Shibata-Koyama M., Isoda Y., Iida S., Masuda K., Satoh M., Kato K. (2011). Structural Basis for Improved Efficacy of Therapeutic Antibodies on Defucosylation of Their Fc Glycans. Genes Cells.

[27] Falconer D.J., Subedi G.P., Marcella A.M., Barb A.W. (2018). Antibody Fucosylation Lowers the FcγRIIIa/CD16a Affinity by Limiting the Conformations Sampled by the N162-Glycan. ACS Chem. Biol..

[28] Okazaki A., Shoji-Hosaka E., Nakamura K., Wakitani M., Uchida K., Kakita S., Tsumoto K., Kumagai I., Shitara K. (2004). Fucose Depletion from Human IgG1 Oligosaccharide Enhances Binding Enthalpy and Association Rate Between IgG1 and FcγRIIIa. J. Mol. Biol..

[29] Coënon L., Rigal E., Courot H., Multrier C., Zemiti S., Lambour J., Pugnière M., De Toledo M., Bossis G., Cartron G. (2024). Generation of Non-Genetically Modified, CAR-like, NK Cells. J. Immunother. Cancer.

[30] Liu Z., Gunasekaran K., Wang W., Razinkov V., Sekirov L., Leng E., Sweet H., Foltz I., Howard M., Rousseau A.-M. (2014). Asymmetrical Fc Engineering Greatly Enhances Antibody-Dependent Cellular Cytotoxicity (ADCC) Effector Function and Stability of the Modified Antibodies. J. Biol. Chem..

[31] Smith P., DiLillo D.J., Bournazos S., Li F., Ravetch J.V. (2012). Mouse Model Recapitulating Human Fcγ Receptor Structural and Functional Diversity. Proc. Natl. Acad. Sci. USA.

[32] Oganesyan V., Damschroder M.M., Leach W., Wu H., Dall’Acqua W.F. (2008). Structural Characterization of a Mutated, ADCC-Enhanced Human Fc Fragment. Mol. Immunol..

[33] Yanaka S., Yogo R., Inoue R., Sugiyama M., Itoh S.G., Okumura H., Miyanoiri Y., Yagi H., Satoh T., Yamaguchi T. (2019). Dynamic Views of the Fc Region of Immunoglobulin G Provided by Experimental and Computational Observations. Antibodies.

[34] Richards J.O., Karki S., Lazar G.A., Chen H., Dang W., Desjarlais J.R. (2008). Optimization of Antibody Binding to FcγRIIa Enhances Macrophage Phagocytosis of Tumor Cells. Mol. Cancer Ther..

[35] Mimoto F., Igawa T., Kuramochi T., Katada H., Kadono S., Kamikawa T., Shida-Kawazoe M., Hattori K. (2013). Novel Asymmetrically Engineered Antibody Fc Variant with Superior FcγR Binding Affinity and Specificity Compared with Afucosylated Fc Variant. mAbs.

[36] Mimoto F., Kadono S., Katada H., Igawa T., Kamikawa T., Hattori K. (2014). Crystal Structure of a Novel Asymmetrically Engineered Fc Variant with Improved Affinity for FcγRs. Mol. Immunol..

[37] Gunasekaran K., Pentony M., Shen M., Garrett L., Forte C., Woodward A., Ng S.B., Born T., Retter M., Manchulenko K. (2010). Enhancing Antibody Fc Heterodimer Formation through Electrostatic Steering Effects. J. Biol. Chem..

[38] Jebamani P., Sriramulu D.K., Lee S.-G. (2023). Residue Interaction Network and Molecular Dynamics Simulation Study on the Binding of S239D/I332E Fc Variant with Enhanced Affinity to FcγRIIIa Receptor. J. Mol. Graph. Model..

[39] Ma B. (2021). Correlation of N-Glycan Dynamics and Interaction Network with Allosteric Antigen Binding and Fc Receptor Recognition. Explor. Immunol..

[40] Jebamani P., Jo M., Park S., Kim S., Jung S.T., Lee S.-G., Wu S. (2025). Design of an Fc Mutation to Abrogate Fcγ Receptor Binding Based on Residue Interaction Network Analysis. ACS Synth. Biol..

[41] Kralj S., Hodošček M., Podobnik B., Kunej T., Bren U., Janežič D., Konc J. (2021). Molecular Dynamics Simulations Reveal Interactions of an IgG1 Antibody With Selected Fc Receptors. Front. Chem..

[42] Kutlu A., Çapkın E., Adacan K., Yüce M. (2024). Fc–FcγRI Complexes: Molecular Dynamics Simulations Shed Light on Ectodomain D3′s Potential Role in IgG Binding. ACS Omega.

[43] Zhao J., Nussinov R., Ma B. (2019). Antigen Binding Allosterically Promotes Fc Receptor Recognition. mAbs.

[44] Lazar G.A., Dang W., Karki S., Vafa O., Peng J.S., Hyun L., Chan C., Chung H.S., Eivazi A., Yoder S.C. (2006). Engineered Antibody Fc Variants with Enhanced Effector Function. Proc. Natl. Acad. Sci. USA.

[45] Coënon L., Geindreau M., Ghiringhelli F., Villalba M., Bruchard M. (2024). Natural Killer Cells at the Frontline in the Fight against Cancer. Cell Death Dis..

[46] Clementel D., Del Conte A., Monzon A.M., Camagni G.F., Minervini G., Piovesan D., Tosatto S.C.E. (2022). RING 3.0: Fast Generation of Probabilistic Residue Interaction Networks from Structural Ensembles. Nucleic Acids Res..

[47] Piovesan D., Minervini G., Tosatto S.C.E. (2016). The RING 2.0 Web Server for High Quality Residue Interaction Networks. Nucleic Acids Res..

[48] Frank M., Walker R.C., Lanzilotta W.N., Prestegard J.H., Barb A.W. (2014). Immunoglobulin G1 Fc Domain Motions: Implications for Fc Engineering. J. Mol. Biol..

[49] Kremer P.G., Lampros E.A., Blocker A.M., Barb A.W. (2024). One N-Glycan Regulates Natural Killer Cell Antibody-Dependent Cell-Mediated Cytotoxicity and Modulates Fc γ Receptor IIIa/CD16a Structure. eLife.

[50] Isoda Y., Yagi H., Satoh T., Shibata-Koyama M., Masuda K., Satoh M., Kato K., Iida S. (2015). Importance of the Side Chain at Position 296 of Antibody Fc in Interactions with FcγRIIIa and Other Fcγ Receptors. PLoS ONE.

[51] Nimmerjahn F., Ravetch J.V. (2005). Divergent Immunoglobulin G Subclass Activity Through Selective Fc Receptor Binding. Science.

[52] Sakae Y., Satoh T., Yagi H., Yanaka S., Yamaguchi T., Isoda Y., Iida S., Okamoto Y., Kato K. (2017). Conformational Effects of N-Glycan Core Fucosylation of Immunoglobulin G Fc Region on Its Interaction with Fcγ Receptor IIIa. Sci. Rep..

[53] Kiyoshi M., Caaveiro J.M.M., Kawai T., Tashiro S., Ide T., Asaoka Y., Hatayama K., Tsumoto K. (2015). Structural Basis for Binding of Human IgG1 to Its High-Affinity Human Receptor FcγRI. Nat. Commun..

