# Supplementary material for: Microsecond Dynamics of Fc–CD16a Recognition: Impact of Mutations, Core Fucosylation, and Fc Asymmetry
Source: Antibodies (Basel). 2026 Feb 23;15(1):17. doi: 10.3390/antib15010017 (PMC12937375; doi:10.3390/antib15010017)
Supplement: Supplementary file 1 [file antibodies-15-00017-s001.zip › antibodies-4060818-supplementary.pdf]

Microsecond Dynamics of Fc–CD16a Recognition: Impact of Mutations, Core Fucosylation, and Fc Asymmetry.

Table of Contents

1 Detailed Computational procedures 3
1.1 Homology modelling of Human CD16aV158 in complex with IgG1-Fc 3
1.2 Molecular dynamics (MD) simulations 4
1.3 Analysis of Molecular Dynamics Trajectories 5
1.3.1 Initial Processing and Validation of MD Trajectories 5
1.3.2 Interaction Network Analysis During Dynamics 6
1.3.3 Statistical Analysis 7
1.4 General evaluation of molecular dynamics simulations 7
2 Protein Sequences, Mutations and Glycan Compositions 9
2.1 Protein Sequences 9
Fc-af 9
Fc4m-af 9
Fc4m-f 10
FcAs-f 10
2.2 Mutations at Key Positions of Fc and CD16a 11
2.3 Glycan Compositions According to IUPAC Standards 11
Fc 11
CD16a 11
3 Overview of MD systems 13
Table S1. 13
4. General Analysis of molecular dynamics simulations 14
4.1 RMSD of Protein chains 14
Figure S1 : 14
4.2 RMSF of Protein chains 15
Figure S2 : 15
4.3 Radius of Gyration Analysis of Protein Complexes 15
Figure S3 : 15
4.4 Average values of RMSD, RMSF for each protein chain and Rg for protein complexes 16
Figure S4a : 16
Figure S4b : 16
Figure S4c : 16

4.5 Solvent Accessible Surface Area of protein complexes or glycan chains 17
Figure S5a : 17
Figure S5b : 17
4.6 Per-atom RMSF of glycan residues across Fc glycovariants... 18
Figure S6: 18
5 Noncovalent bond interactions between amino acids 19
Table S2. 19
Table S3. 20
Table S4-1. 21
Table S4-2. 21
Table S5-1. 22
Table S5-2. 22
6 Intermolecular van der Waals interaction 24
Figure S7 : 26
Table S6-1. 27
Table S6-2. 28
7 Hydrogen Bond Interaction Between Protein Residues and Glycan Sugars 29
Figure S8 : 29
Table S8-1. 31
Table S8-2. 32
8 Hydrogen bond interaction between sugars of glycan chains. 33
Figure S9 : 34
Table S9-1. 35
Table S9-2. 35
9 Intermolecular Interactions at the CH3–CH3 Interface of Fc Variants.. 36
Figure S10 : 36
10 The local hydrophobic patch with Trp236 in the FcAs-f complex 37
Figure S11 : 37
11 Pairwise MM/GBSA residue interaction enthalpies for the Fc–CD16a complexes 38
Figure S12 : 38

1 Detailed Computational procedures

1.1 Homology modelling of Human CD16aV158 in complex with IgG1-Fc

To investigate the impact on the interaction network between Fc and CD16a of DFTE or asymmetric mutations and the presence of fucose on both chains of Fc, four distinct complexes (Fc-af, Fc4m-af, Fc4m-f and FcAs-f) were engineered and subjected to 1µs molecular dynamics simulations across four replicas. Functional polymorphisms of CD16a have been identified at position 158 (V/F), with the 158V variant exhibiting higher affinity for IgG. This enhanced binding capacity is associated with improved ADCC efficiency and better clinical responses to therapeutic antibodies such as rituximab.

To construct the initial model, which lacks both mutations and fucose (Fc-af), we first aligned the sequences against known proteins in the Protein Data Bank. This included the human Immunoglobulin Heavy Constant Gamma 1 (UniProt entry: P01857) and the soluble form of the human Low Affinity Immunoglobulin Gamma Fc Region Receptor III-A, FcγRIIIa (UniProt entry: P08637). Following these alignments, the structure of the human nonfucosylated Fc in complex with the bis-glycosylated soluble form of Fc gamma receptor IIIa was selected as the template (PDB ID: 3AY4)(20). Based on this template, the protein complex was constructed using Modeller(27), comprising two chains for Fc (residues Cys 229-Leu 443 for chain A and Cys 229-Ser 444 for chain B) and one chain for CD16a featuring the Val 158 variant (CD16a, residues Leu 5-Gln 174, chain C).

The model includes seven disulfide bridges (Cys 229:A- Cys 229:B; Cys 261:A- Cys 321:A; Cys 367:A- Cys 425:A; Cys 261:B- Cys 321:B; Cys 367:B- Cys 425:B; Cys 110:C- Cys 154:C; CYS29:C- CYS71:C). For chain C, the sequence G31-T40 was reconstructed using the Prepare Protein module in BIOVIA Discovery Studio (Biovia Discovery Studio Modeling Environment, release 2021). An alternative template, the crystal structure of GASDALIE IgG1 Fc in complex with CD16a (PDB ID: 5D6D), was not utilized due to the absence of several sugars on CD16a(19).

All glycans from the PDB structure template 3AY4 were retained in the model(28). On chain A and B, glycans on Asn297 Car A and Car B, respectively, are composed as follows: beta-D-galactopyranose-(1-4)-2-acetamido-2-deoxy-beta-D-glucopyranose-(1-2)-alpha-D-mannopyranose-(1-6)-[2-acetamido-2-deoxy-beta-D-glucopyranose-(1-2)-alpha-D-mannopyranose-(1-3)]beta-D-mannopyranose-(1-4)-2-acetamido-2-deoxy-beta-D-glucopyranose-(1-4)-2-acetamido-2-deoxy-beta-D-glucopyranose.

On chain C, the glycan Car C linked to Asn162 is 2-acetamido-2-deoxy-beta-D-glucopyranose-(1-2)-alpha-D-mannopyranose-(1-3)-[2-acetamido-2-deoxy-beta-D-glucopyranose-(1-2)-alpha-D-mannopyranose-(1-6)]beta-D-mannopyranose-(1-4)-2-acetamido-2-deoxy-beta-D-glucopyranose-(1-4)-[alpha-L-fucopyranose-(1-6)]2-acetamido-2-deoxy-beta-D-glucopyranose and Car D on Asn45 is beta-D-mannopyranose-(1-4)-2-acetamido-2-deoxy-beta-D-glucopyranose-(1-4)-2-acetamido-2-deoxy-beta-D-glucopyranose.

To construct the afucosylated Fc4m-af model with four mutations (S239D, H268F, S324T, I332E) on chains A and B of the Fc protein, twenty homology models were generated via Modeller, integrated within BIOVIA Discovery Studio. The evaluation of these models employed both the PDF score, specific to Modeller, and the QMEAN scoring system(29) which conducts the selection of the best model (PDF score 8589. , QMEAN score 0.86). Consistent with the initial model Fc-af, Fc4m-af also features the same disulfide bridges, the reconstructed loop between G31 and T40 residues, and glycan composition (See Sup Info).

For molecular dynamics simulations, PDB files of both Fc-af and Fc4m-af complexes, including proteins and glycan chains, were prepared using the CHARMM-GUI facilities(28, 30), notably the Glycan

Modeler(31) (https://www.charmm-gui.org/). The third protein complex, Fc4m-f, which features the four mutations (S239D, H268F, S324T, I332E) along with the addition of a α-fucose on both chains of Fc, was generated by modifying the Fc4m-af file using the CHARMM-GUI interface. To accommodate the two additional fucoses without causing steric clashes with seven carbohydrates N-linked attached to Asn162 on chain C, referred to as Car C, this glycan was reconstructed using the Glycan Builder tool on CHARMM-GUI.

The PDB structure 3WN5 was used as a template to construct the asymmetric fucosylated Fc structure in complex with CD16a (FcAs-f model). To ensure consistency with the chain assignments used in the other Fc complexes, the original chain identifiers from the PDB file were manually reassigned, such that chain A of the model corresponds to the heavy chain bearing S239M. This model includes eight substitutions on chain A (L234Y, L235Y, G236W, S239M, H268D, S298A, A327D, and Y349C) and nine distinct mutations on chain B (D270E, K326D, A330K, K334E, D356C, T366S, L368A, and Y407V). Additionally, CPAPEY and CPAP residues were appended to the N-terminus of chains A and B, respectively, using MODELLER. For chain C, corresponding to CD16a, an aspartic acid residue (D4) was added to the N-terminus, while residues E36 and D37 were reconstructed using MODELLER. The FcAs-f model retained the same disulfide bridges and glycan composition as the initial Fc-af model, with the addition of a fucose on glycan sites of Fc chains A and B. These sites are β-fucosylated in the FcAs-f complex to respect the asymmetric engineering of Fc described in the 3WN5 structure. PDB files of the FcAs-f complex, including both protein and glycan chains, were prepared for molecular dynamics simulations using CHARMM-GUI facilities (Table S1, Supp Info).

1.2 Molecular dynamics (MD) simulations

Using the CHARMM-GUI web site, each pdb complex was solvated in a cubic periodic box with explicit TIP3P water molecules, extending 15 Å beyond the macromolecules. The systems were neutralized with counterions using 0.1 M of NaCl, applied through a Monte Carlo method. Details regarding the number of atoms for each system component are provided in Supplementary Table S1.

Atomic interactions for both the protein and glycans were modeled using the all-atom CHARMM36m force field, with CHARMM-GUI facilitating the translation of parameters for compatibility with the GROMACS 2022.2 simulation environment(32–34). CHARMM36m includes dedicated parameters for carbohydrates, allowing proteins and glycans to be treated within a single, internally consistent force-field framework. To relax the structures, the minimization phase employed a steepest descent algorithm for 5000 steps until a force tolerance of 1000 kJ/mol/nm was achieved. Non-bonded interactions were managed with a Verlet cutoff scheme, implementing a force-switch modification for van der Waals forces between 1.0 and 1.2 nm, and using Particle Mesh Ewald for electrostatic interactions with a corresponding cutoff of 1.2 nm(35, 36). Initial structure preparation included the application of positional restraints on the protein backbone and sidechains with force constants of 400 and 40 kJ/mol/nm^2, respectively, and dihedral restraints with a force constant of 4 kJ/mol/nm^2. Subsequently, the system underwent an equilibration phase under constant number, volume, and temperature (NVT) conditions over 10 ns, with a 1 fs integration timestep. Temperature regulation was facilitated by the Nosé-Hoover thermostat with a coupling time constant of 1 ps, maintaining the solute and solvent at 303.15 K. For the production phase, the simulation was conducted in the NPT ensemble (constant number of particles, pressure, and temperature) and extended to 1 µs trajectories, performed as ten consecutive 100 ns segment using a 2 fs integration timestep. Each segment was initialized from the final coordinates and velocities of the preceding one. The Nosé-Hoover thermostat

was utilized to maintain a consistent temperature of 303.15 K for both the solute and solvent with a coupling constant of 1 ps, ensuring the system remained at the desired thermal state throughout the simulations. Pressure was regulated at 1 bar using the Parrinello-Rahman barostat with a coupling time constant of 5 ps and a compressibility of 4.5e-5 bar^-1. Throughout both equilibration and production phases, all hydrogen-containing bonds were constrained using the LINCS algorithm to permit a larger integration timestep. Initial velocities, following a Maxwellian distribution at 303.15 K, were generated at the commencement of the equilibration and were sustained through the continuation option into the production phase. During the production phase, trajectory snapshots were captured every 200 ps, providing a total of 5000 frames (10x500) per system for subsequent analysis. To ensure robust sampling and account for statistical variations, four independent replica simulations were conducted for each protein complex, totaling sixteen 1-microsecond dynamics runs. Each replica originated from distinctly equilibrated starting conditions, differing only in the initial velocity distributions, using the GROMACS framework.

The molecular dynamics calculations were performed on the Jean Zay supercomputer at IDRIS, utilizing its GPU capabilities to efficiently parallelize and execute the four replica simulations simultaneously on a single NVIDIA Tesla V100 card. This approach enabled the concurrent processing of all four replicas, each spanning 1 microsecond of simulation time, thereby optimizing computational resources and time.

1.3 Analysis of Molecular Dynamics Trajectories

Protein complexes from the molecular dynamics trajectories were visualized using PyMOL and analyzed with a suite of tools, including the standard analysis utilities available in the GROMACS package. For advanced interrogation of protein-protein interactions, the Residue Interaction Network Generator (RING)(37, 38), a PyMOL plugin, was employed to ascertain the frequencies of non-covalent interactions between residues during molecular dynamics. Then, custom Python scripts were utilized, operating within an Anaconda environment. The scripting facilitated the targeted extraction and quantification of specific non-bonded interactions between the protein chains and the carbohydrate moieties of the glycans, as well as interactions within the glycans themselves. The environment setup in Anaconda for molecular trajectory analysis involved a sequence of targeted installations which encompassed libraries for molecular visualization, image processing, data analysis, and interactive development, including an open-source version of PyMOL (Schrodinger, LLC) for molecular visualization. The environment was built with MoviePy, ImageIO, Pillow for image processing, and scientific computing tools such as Matplotlib(39), RDKit, Pandas, PyTest, and NumPy(40) for data analysis. Additional installations like Yarn, NodeJS, and Pip supported the development environment, while JupyterLab, Ipywidgets, ASE, ParmEd, PyMatGen, MDTraj(41), Py3Dmol, Biopython, MDAnalysis(42), Seaborn(43), and IPython enhanced the analytical and visualization approach. Jupyter extensions for NGLview(44) were enabled for seamless integration in Jupyter notebooks. This setup provided a robust framework for performing high-resolution analyses of molecular dynamics trajectories, facilitating both the exploration of protein-protein interactions and the detailed study of glycan dynamics.

1.3.1 Initial Processing and Validation of MD Trajectories

At first, GROMACS tools were employed to merge trajectories, reposition molecules to the center of the periodic box, rectify any discontinuities in molecular structures, and remove translational and rotational movements of the proteins. Molecular dynamics simulations spanning 1 microsecond were conducted for each of the protein complexes: Fc-af, Fc4m-af, Fc4m-f, and FcAs-f across four replicas. However, to ensure that each system reached a stable state, the first 100 nanoseconds of each trajectory were excluded from analysis to account for observed conformational rearrangements, ensuring a stabilized representation of the Fc-CD16a binding interface. This precaution was particularly crucial for the Fc4m-f complex, which underwent significant modifications such as glycan reconstruction on chain C of CD16a and the addition of fucose to chains A and B of Fc. The stabilization of these configurations was visually confirmed by approximately 50 nanoseconds. Consequently, the remaining 900 nanoseconds of each trajectory were analyzed to calculate observables such as root-mean-square deviation (RMSD), radius of gyration (Rg), root-mean-square fluctuations (RMSF) using the MDAnalysis toolkit.

RMSD of the coordinates of each protein's Cα atoms was computed as a function of time in reference to the equilibrated initial structure from the production run for confirming simulation convergence. For the position of heavy atoms, the equilibrated initial structure is strictly identical to the CHARMM-GUI provided structure. These metrics were chosen to provide insight into the dynamic behavior of the protein structures throughout the simulation period. The Rg, reflecting the compactness of the protein structures, was computed based on the Cα atoms for each frame. RMSF analyses were performed, offering a fluctuation profile on the protein's Cα positions as a function of the amino acid residue index, and providing a nuanced view of the flexibility within the protein structures. The average and standard deviation of each property were calculated across the four replicas for each protein system. For every system, monosaccharide flexibility was quantified as the root-mean-square fluctuation of the anomeric C1 atom.

1.3.2 Interaction Network Analysis During Dynamics

The interaction networks throughout the molecular dynamics simulations were analyzed using multiple tools. The Residue Interaction Network Generator (RING) 2.0, implemented as a PyMOL plugin, was used to map non-covalent interactions including hydrogen bonds, van der Waals interactions, π-π stacking, π-cation interactions, ionic salt bridges, and disulfide bonds across all frames of a trajectory(37, 38). Custom Python scripts further analyzed the frequency and duration of both intra- and inter-chain residue interactions. In this study, hydrogen and ionic bonds were examined in detail, with interactions reported for the 900 ns of dynamics using a 10% cutoff (lasting at least 90 ns). Hydrogen and ionic bonds between protein residues were quantified at the inter-chain level using MDAnalysis via Python scripts and Jupyter notebooks. Additionally, hydrogen bonds between proteins and glycans, as well as intra- and inter-glycan moieties, were analyzed in this study. For glycan interactions, the cumulative duration of hydrogen bonds was calculated and reported, distinguishing interactions between specific residues and individual sugar moieties, as well as between one sugar and another.

Additionally, Solvent Accessible Surface Area (SASA) calculations were performed using GROMACS to understand the variation of the exposure of both the protein and glycan surfaces to the solvent (Figure S10). These measurements were conducted for the complete protein complexes with all glycans, and separately for the individual glycan chains. The SASA analysis provided insights into the dynamic behavior and structural integrity of the Fc complexes, showing no significant differences in solvent exposure between the complexes.

1.3.3 Statistical Analysis

We analyzed duration differences across four Fc–CD16a complexes (Fc-af, Fc4m-af, Fc4m-f, FcAs-f), each sampled by 4 independent MD replicas (900 ns). For protein–protein, we considered hydrogen bonds, electrostatic (ionic) contacts, and van der Waals contacts. For protein–glycan and glycan–glycan, we considered hydrogen bonds. For cross-complex inference, we retained only interaction pairs observed in ≥ 2 complexes. For descriptive “unique” summaries, we also reported pairs present in exactly one complex, optionally requiring a mean-duration cutoff (typically ≥ 90 ns).

For each inter-residue contact, contact lifetimes from the four replicas of each complex were exported in long format (one row per contact, complex, and replica). For a given residue pair, this yielded a small distribution of lifetimes for each complex. Pairwise comparisons between complexes were then performed using Welch’s two-sided t-test with unequal variances (SciPy ttest_ind, equal_var=False) on these per-complex lifetime distributions. We report group means, mean differences (Δ), and p-values with $\alpha = 0.05$. Given the descriptive, atlas-oriented nature of this study and the large number of contacts examined, p-values were used as a filtering criterion rather than for formal discovery. We did not apply multiple-testing corrections; instead, significance ($p < 0.05$) was combined with physically stringent filters to limit false positives arising from noise. Specifically, we only discuss in the main text those contacts that (i) reach a replicate-averaged lifetime (e.g. ≥ 90 ns) in at least one complex and (ii) exhibit a significant difference between complexes (Welch’s t-test, $p < 0.05$).Software. Analyses were performed in Python using pandas (data handling) and SciPy (Welch’s t-tests). Excel I/O and user inputs (thresholds, file selection) were handled via Tkinter dialogs.

1.4 General evaluation of molecular dynamics simulations

To ensure the proper progression during the 16 molecular dynamics simulations, several classical parameters were investigated. First, the structural conformations and backbone flexibility of the amino acid chains were studied using standard methods such as Root Mean Square Deviation (RMSD), Root Mean Square Fluctuation (RMSF) and the Radius of Gyration (Rg) (Figure S1-3). The RMSD analysis was performed for each replica to monitor the stability of each chain of the protein complexes over the 900 ns simulations. Figure S1 illustrates the RMSD fluctuations for each replica (D1 to D4), indicating small variations between the different replicated simulations. Generally, the fluctuations range from 1 Å to 4 Å, suggesting that the complexes exhibit moderate flexibility while maintaining their overall conformation. Figure S4a presents the average RMSD for Fc chains A and B, as well as for CD16a, revealing relatively comparable stability among the molecular complexes. The average RMSD values are quite low, ranging from 1 Å to 3 Å, although some variations are noted.

The RMSF analysis (Figure S2) indicates that all complexes exhibit moderate flexibility, with RMSF values ranging from 1 Å to 4 Å. This suggests that while there are inherent movements within the protein backbones, the overall conformations are retained. In Figure S4b, the average RMSF values reveal that the complexes exhibit comparable flexibility across the amino acid residues and that the mutations in Fc4m-af, Fc4m-f and FcAs-f do not drastically alter the flexibility profile compared to the Fc-af complex (Figure S4b).

The Radius of Gyration (Rg) profiles in Figure S3 indicate the overall compactness of the protein complexes over the simulation time. The Rg values for the four complexes generally fluctuate between 31 nm and 34 nm, suggesting a stable and compact structure throughout the 900 ns simulations. The

individual traces for each replica show consistent Rg values, indicating reproducibility across different simulation runs. Figure S4c, which presents the average Rg values, further supports these findings.

These analyses did not show significant differences between the dynamics of the different protein complexes, suggesting that structural stability was maintained across all variants, confirming the consistency and reliability of the molecular dynamic simulations conducted. Throughout the molecular dynamics simulations, the potential and kinetic energies were monitored to ensure the stability and equilibration of the system (data not shown).

Finally, the conformational behavior of the Fc N-glycans was evaluated through residue-level RMSF analysis across the 16 replicas (Figure S6). The GlcNAc cores and the Man3 backbone exhibit low and reproducible RMSF values, indicating rapid convergence and confinement within a stable motional basin after equilibration. The terminal antennae display broader fluctuations, as expected from their intrinsic flexibility, yet their profiles remain consistent across replicas. Core fucosylation has a clear impact on the CAR C glycan, which display a slightly increased RMSF in the fucosylated complexes. These patterns confirm the convergence of glycan dynamics while capturing localized, variant-dependent differences relevant for subsequent interaction analyses.

MMPBSA : Pairwise residue interaction energies between the Fc fragment (FcA and FcB) and CD16a were evaluated using the molecular mechanics/generalized Born surface area (MM/GBSA) approach implemented in gmx_MMPBSA[1]. Post processing was carried out on the 1 µs production trajectories of the four Fc–CD16a complexes (Fc-af, Fc4m-af, Fc4m-f and FcAs-f). A generalized Born implicit solvent model with the default gmx_MMPBSA parameters was employed. Residue pair decomposition was performed with decomp=4, defining the solute index group as FcA, FcB and CD16a and the solvent as water and ions. The print_reslist option was used to restrict the output to interacting FcA–CD16a and FcB–CD16a residue pairs. Trajectory frames had been stored every 200 ps, yielding 5000 frames over the 1 µs production run. For MM/GBSA analysis, 91 snapshots per trajectory were extracted from frames 500 to 5000 using a stride of 50 frames, which corresponds to configurations sampled every 10 ns from 100 to 1000 ns of the simulation. Only enthalpic contributions (molecular mechanics and solvation) were considered in the pairwise decomposition, so the reported values represent relative interaction enthalpies rather than absolute binding free energies. The print_reslist option was used to restrict the output to interacting FcA–CD16a and FcB–CD16a residue pairs, as shown in Figs. 2 and 3.

2 Protein Sequences, Mutations and Glycan Compositions

Overview of Fc variants investigated in this study, summarizing chain-specific mutations and Fc glycan fucosylation status.

**Fc variant**	**Fc chain A mutations**	**Fc chain B mutations**	**Fc glycan at Asn297 (CAR A / CAR B)**
**Fc-af**	None (wild-type)	None (wild-type)	Afucosylated / Afucosylated
**Fc4m-af**	S239D, H268F, S324T, I332E	S239D, H268F, S324T, I332E	Afucosylated / Afucosylated
**Fc4m-f**	S239D, H268F, S324T, I332E	S239D, H268F, S324T, I332E	Core-fucosylated (α1–6) / Core-fucosylated (α1–6)
**FcAs-f**	L234Y, L235Y, G236W, S239M, H268D, S298A, A327D, Y349C	D270E, K326D, A330K, K334E, D356C, T366S, L368A, Y407V	Core-fucosylated (β-linked) / Core-fucosylated (β-linked)

2.1 Protein Sequences

Fc-af

Fc ChainA 229-443
CPAPELLGGPSVFLFPPKPKDTLMISRTPEVTCVVVDVSHEDPEVKFNWYVDGVEVHNAKTKPREEQYNSTYRVVSVLTVLHQD
WLNGKEYKCKVSNKALPAPIEKTISKAKGQPREPQVYTLPPSRDELTKNQVSLTCLVKGFYPSDIAVEWESNGQPENNYKTTPPV
LDSDGSFFLYSKLTVDKSRWQQGNVFSCSVMHEALHNHYTQKSLSL

ChainB 229-444
CPAPELLGGPSVFLFPPKPKDTLMISRTPEVTCVVVDVSHEDPEVKFNWYVDGVEVHNAKTKPREEQYNSTYRVVSVLTVLHQD
WLNGKEYKCKVSNKALPAPIEKTISKAKGQPREPQVYTLPPSRDELTKNQVSLTCLVKGFYPSDIAVEWESNGQPENNYKTTPPV
LDSDGSFFLYSKLTVDKSRWQQGNVFSCSVMHEALHNHYTQKSLSLS

CD16a ChainC 5-174
LPKAVVFLEPQWYRVLEKDSVTLKCQGAYSPEDQSTQWFHNESLISSQASSYFIDAATVDDSGEYRCQTQLSTLSDPVQLEVHIG
WLLLQAPRWVFKEEDPIHLRCHSWKNTALHKVTYLQNGKGRKYFHHNSDFYIPKATLKDSGSYFCRGLVGSKNVSSETVQITITQ

Disulfide bridges Fc-af
C229-A_C229-B, C261-A_C321-A, C367-A_C425-A, C261-B_C321-B, C367-B_C425-B, C29-C_C71-C, C110-C_C154-C

Fc4m-af

Fc ChainA 229-443
CPAPELLGGPDVFLFPPKPKDTLMISRTPEVTCVVVDVSFEDPEVKFNWYVDGVEVHNAKTKPREEQYNSTYRVVSVLTVLHQD
WLNGKEYKCKVTNKALPAPEEKTISKAKGQPREPQVYTLPPSRDELTKNQVSLTCLVKGFYPSDIAVEWESNGQPENNYKTTPP
VLDSDGSFFLYSKLTVDKSRWQQGNVFSCSVMHEALHNHYTQKSLSL

ChainB 229-443

CPAPELLGGPDVFLFPPKPKDTLMISRTPEVTCVVVDVSFEDPEVKFNWYVDGVEVHNAKTKPREEQYNSTYRVVSVLTVLHQD
WLNGKEYKCKVTNKALPAPEEKTISKAKGQPREPQVYTLPPSRDELTKNQVSLTCLVKGFYPSDIAVEWESNGQPENNYKTTPP
VLDSDGSFFLYSKLTVDKSRWQQGNVFSCSVMHEALHNHYTQKSLSL

CD16a ChainC 5-174
LPKAVVFLEPQWYRVLEKDSVTLKCQGAYSPEDNSTQWFHNESLISSQASSYFIDAATVDDSGEYRCQTNLSTLSDPVQLEVHIG
WLLLQAPRWVFKEEDPIHLRCHSWKNTALHKVTYLQNGKGRKYFHHNSDFYIPKATLKDSGSYFCRGLVGSKNVSSETVNITITQ

Disulfide bridges Fc4m-af
C229-A_C229-B, C261-A_C321-A, C367-A_C425-A, C261-B_C321-B, C367-B_C425-B, C29-C_C71-C, C110-C_C154-C

Fc4m-f

Fc ChainA 229-443
CPAPELLGGPDVFLFPPKPKDTLMISRTPEVTCVVVDVSFEDPEVKFNWYVDGVEVHNAKTKPREEQYNSTYRVVSVLTVLHQD
WLNGKEYKCKVTNKALPAPEEKTISKAKGQPREPQVYTLPPSRDELTKNQVSLTCLVKGFYPSDIAVEWESNGQPENNYKTTPP
VLDSDGSFFLYSKLTVDKSRWQQGNVFSCSVMHEALHNHYTQKSLSL

ChainB 229-443
CPAPELLGGPDVFLFPPKPKDTLMISRTPEVTCVVVDVSFEDPEVKFNWYVDGVEVHNAKTKPREEQYNSTYRVVSVLTVLHQD
WLNGKEYKCKVTNKALPAPEEKTISKAKGQPREPQVYTLPPSRDELTKNQVSLTCLVKGFYPSDIAVEWESNGQPENNYKTTPP
VLDSDGSFFLYSKLTVDKSRWQQGNVFSCSVMHEALHNHYTQKSLSL

CD16a ChainC 5-174
LPKAVVFLEPQWYRVLEKDSVTLKCQGAYSPEDNSTQWFHNESLISSQASSYFIDAATVDDSGEYRCQTNLSTLSDPVQLEVHIG
WLLLQAPRWVFKEEDPIHLRCHSWKNTALHKVTYLQNGKGRKYFHHNSDFYIPKATLKDSGSYFCRGLVGSKNVSSETVNITITQ

Disulfide bridges Fc4m-f
C229-A_C229-B, C261-A_C321-A, C367-A_C425-A, C261-B_C321-B, C367-B_C425-B, C29-C_C71-C, C110-C_C154-C

FcAs-f

Fc ChainA 229-444

CPAPELLGGPSVFLFPPKPKDTLMISRTPEVTCVVVDVSHEEPEVKFNWYVDGVEVHNAKTKPREEQYNSTYRVVSVLTVLHQD
WLNGKEYKCKVSNDALPKPIEETISKAKGQPREPQVYTLPPSRCELTKNQVSLSCAVKGFYPSDIAVEWESNGQPENNYKTTPPV
LDSDGSFFLVSKLTVDKSRWQQGNVFSCSVMHEALHNHYTQKSLSLS

ChainB 229-444

CPAPEYYWGPMVFLFPPKPKDTLMISRTPEVTCVVVDVSDEDPEVKFNWYVDGVEVHNAKTKPREEQYNATYRVVSVLTVLH
QDWLNGKEYKCKVSNKDLPAPIEKTISKAKGQPREPQVCTLPPSRDELTKNQVSLWCLVKGFYPSDIAVEWESNGQPENNYKTT
PPVLDSDGSFFLYSKLTVDKSRWQQGNVFSCSVMHEALHNHYTQKSLSLS

CD16a ChainC 4-174

DLPKAVVFLEPQWYRVLEKDSVTLKCQGAYSPEDQSTQWFHNESLISSQASSYFIDAATVDDSGEYRCQTQLSTLSDPVQLEVHI
GWLLLQAPRWVFKEEDPIHLRCHSWKNTALHKVTYLQNGKGRKYFHHNSDFYIPKATLKDSGSYFCRGLVGSKNVSSETVQITIT
Q

Disulfide bridges FcAs-f

C232-A_C229-B, C261-A_C321-A, C356-A_C349-B, C367-A_C425-A, C261-B_C321-B, C367-B_C425-B, C26-C_C68-C, C107-C_C151-C

2.2 Mutations at Key Positions of Fc and CD16a

Fc Chain A and Chain B

For Fc-af, the sequence of the human Immunoglobulin Heavy Constant Gamma 1 (IgG1, UniProt P01857) is characterized by the presence of S239, H268, S324, and I332. For Fc4m-af and Fc4m-f, four mutations were introduced to the sequence: S239D, H268F, S324T, I332E.

CD16a Chain C

For all complexes, the sequence of the human Low affinity immunoglobulin gamma Fc region receptor III-A (FcγRIIIa, Uniprot P08637) presents the valine variant at position 158.

Mutational Analysis of FcAs-f

A comparison of the amino acid sequence of one chain from FcAs-f with the Fc-af construct reveals eight substitutions: D270E, K326D, A330K, K334E, D356C, T366S, L368A, and Y407V. In the other chain, nine distinct mutations are observed: L234Y, L235Y, G236W, S239M, H268D, S298A, A327D, Y349C, and T366W. Furthermore, for the C chain corresponding to CD16a, an additional aspartic acid residue (D4) is present at the N-terminal. 3WN5

2.3 Glycan Compositions According to IUPAC Standards

Fc Chain A and B

For **Fc-af** and **Fc4m-af**, based on the PDB structure 3AY4, the two glycans, **Car A** and **Car B**, are composed of GlcNAc(b1-2)Man(a1-3)[Gal(b1-4)GlcNAc(b1-2)Man(a1-6)]Man(b1-4)GlcNAc(b1-4)GlcNAc(b1-)N297.

For **Fc4m-f** and **FcAs-f**, the glycan presents an additional fucose.

Fc4m-f, GlcNAc(b1-2)Man(a1-3)[Gal(b1-4)GlcNAc(b1-2)Man(a1-6)]Man(b1-4)GlcNAc(b1-4)[Fuc(a1-6)]GlcNAc(b1-)N297.

FcAs-f, GlcNAc(b1-2)Man(a1-3)[Gal(b1-4)GlcNAc(b1-2)Man(a1-6)]Man(b1-4)GlcNAc(b1-4)[Fuc(b1-6)]GlcNAc(b1-)N297.

CD16a Chain C

All complexes present the 2 following glycans.

Car C :GlcNAc(b1-2)Man(a1-3)[GlcNAc(b1-2)Man(a1-6)]Man(b1-4)GlcNAc(b1-4)[Fuc(a1-6)]GlcNAc(b1-)N162

Car D : Man(b1-4)GlcNAc(b1-4)GlcNAc(b1-)N45.

3 Overview of MD systems

Table S1.

The simulation box in the MD simulations contained two glycosylated proteins, salt ions and water atoms.

Systems			Fc-af	Fc4m-af	Fc4m-f	FcAs-f
	Nb of atoms in the PDB file		295102	288358	288478	294859
	Time / frame in ps		200.0	200.0	200.0	200.0
	Total frames, production		5000	5000	5000	5000
	Total time in ns		1000	1000	1000	1000
Protein Segids	Segid 1: PROA (chain A)	Nb of Residues:	215	215	215	216
		Nb of Atoms:	3395	3398	3398	3428
	Segid 2: PROB (chain B)	Nb of Residues:	216 from pdb 3AY4 1 additional serine	215	215	216
		Nb of Atoms:	3406	3398	3398	3385
	Segid 3: PROC (chain C)	Nb of Residues:	170	170	170	171
		Nb of Atoms:	2706	2697	2699	2718
Non-Protein Segids:	Segid Car A	Nb of Residues:	8	8	9	9
		Nb of Atoms:	193	193	213	213
	Segid Car B	Nb of Residues:	8	8	9	9
		Nb of Atoms:	193	193	213	213
	Segid Car C	Nb of Residues:	8	8	8	8
		Nb of Atoms:	192	192	192	192
	Segid Car D	Nb of Residues:	3	3	3	3
		Nb of Atoms:	76	76	76	76
	Segid SOLV	Nb of Residues:	94860	92618	92644	94756
		Nb of Atoms:	284580	277854	277932	284268
	Segid IONS	Nb of Atoms:	361	357	357	366

4. General Analysis of molecular dynamics simulations

4.1 RMSD of Protein chains

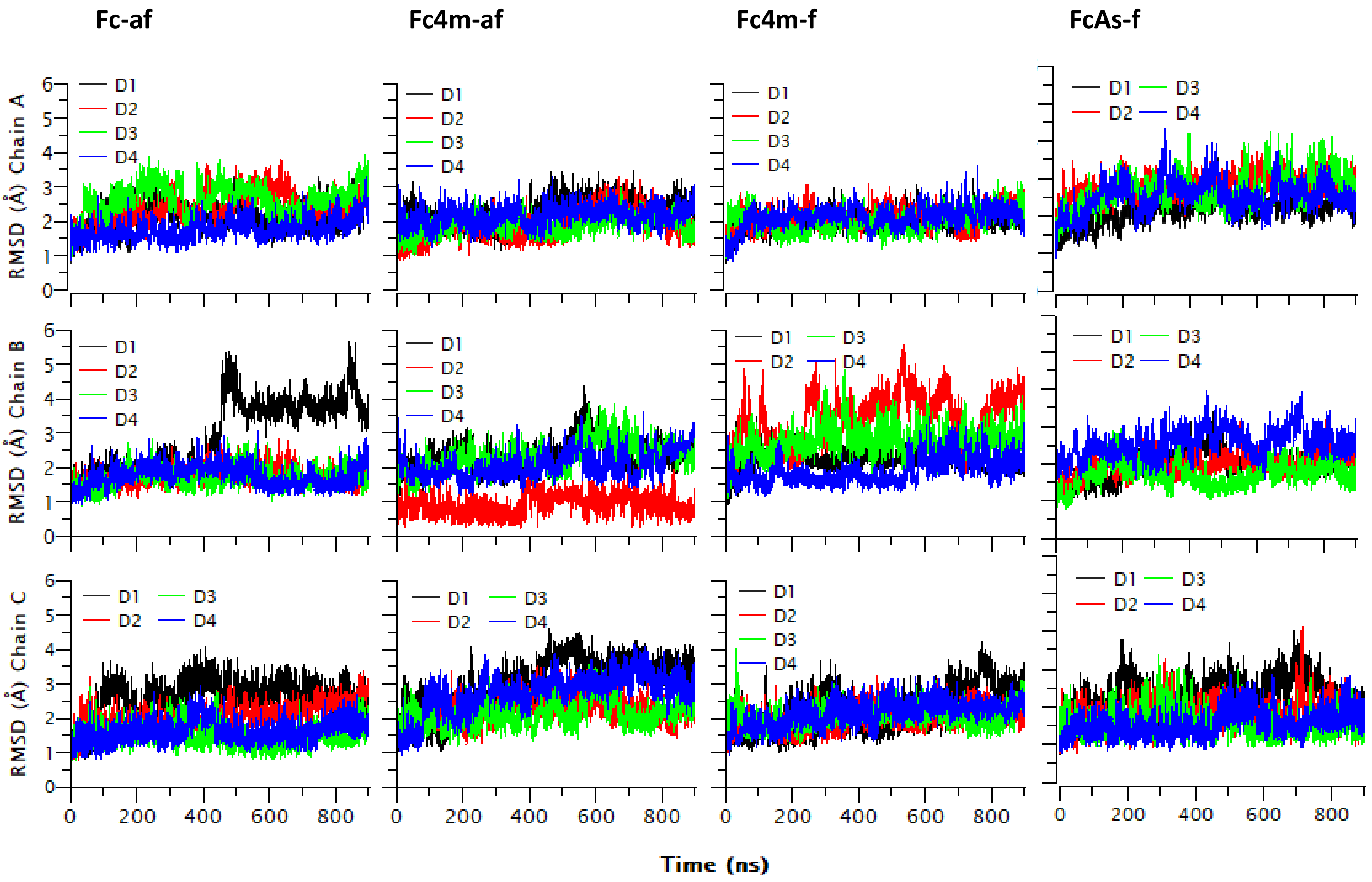

Figure S1 : Cα RMSD of Fc-af, Fc4m-af, Fc4m-f and FcAs-f complexes during the NPT production run of 900 ns for replica 1 (black), replica 2 (red) replica 3 (green) and replica 4 (blue). To identify significant alterations, the root-mean-square deviation (RMSD) of each chain was determined based on the protein's α-carbon (Cα) atoms relative to the terminal equilibrated configuration of each complex which served here as the reference. These computations were conducted across 900 nanoseconds (100 to 1µs) for each replica of the system with the first 100 ns omitted.

4.2 RMSF of Protein chains

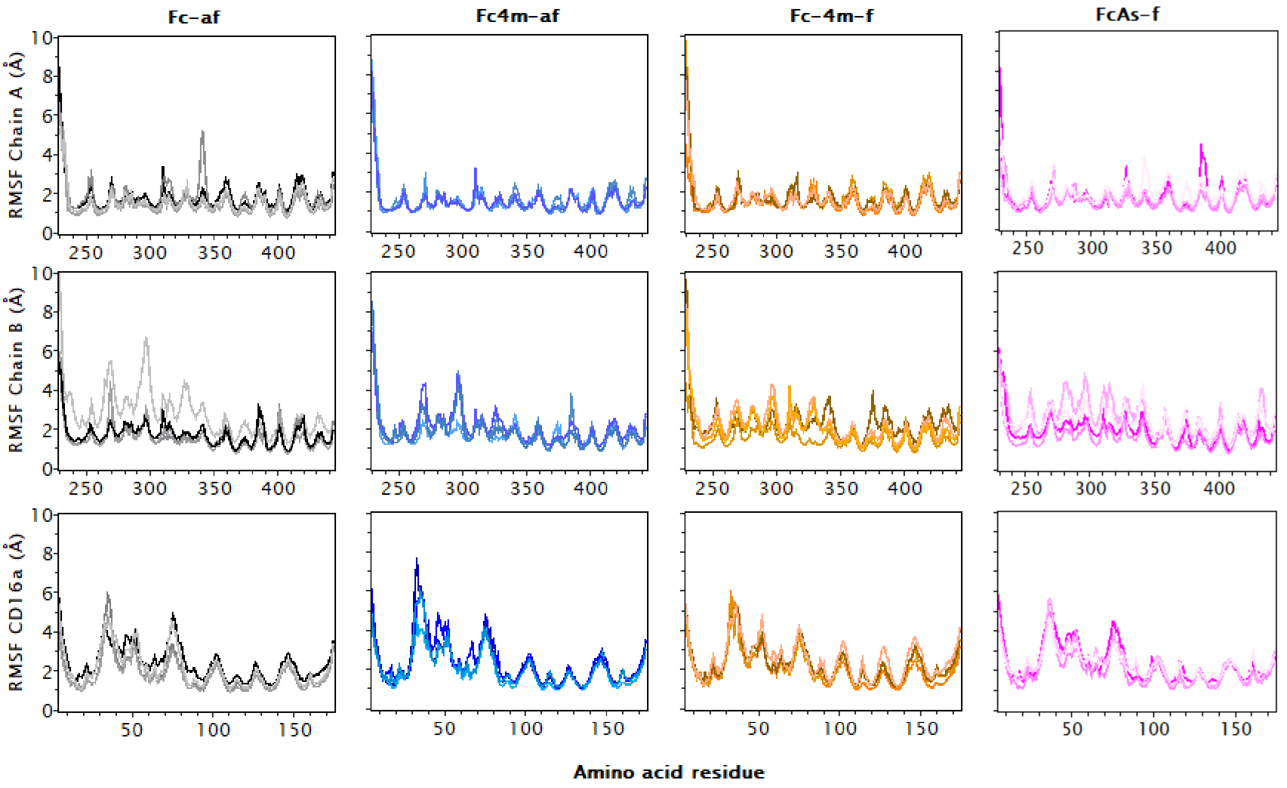

Figure S2 : Root Mean Square Fluctuation (RMSF) for Fc chain A, chain B, and CD16a of each complex: Fc-af (black), Fc4m-af (blue), Fc4m-f (orange), and FcAs-f (pink) across four replicas. RMSF was calculated based on the Cα positions during a 900 ns time production per replica. The RMSF of the Cα atoms provides a measure of the fluctuations in Cartesian coordinates of the protein's backbone, serving as an indicator of backbone flexibility.

4.3 Radius of Gyration Analysis of Protein Complexes

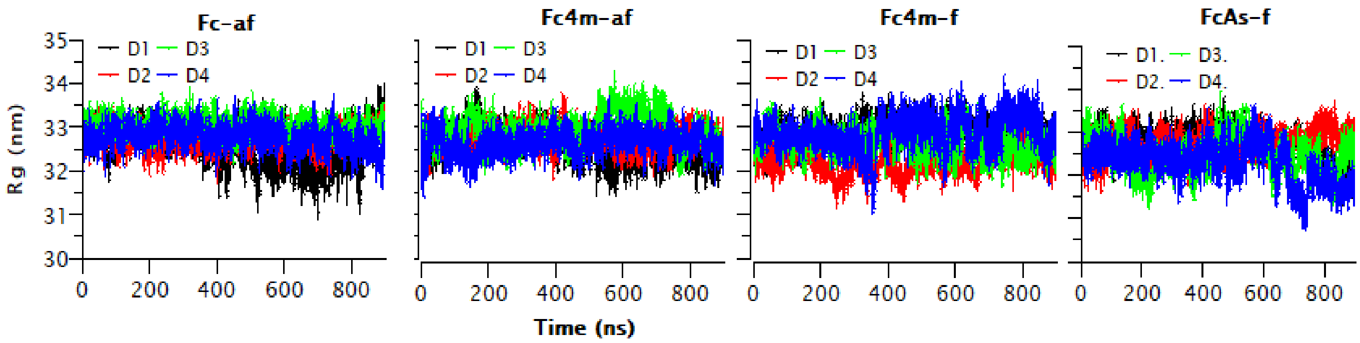

Figure S3 : Radius of gyration (Rg) profiles for the four studied protein complexes Fc-af, Fc4m-af, Fc4m-f and FcAs-f are presented. The Rg was calculated based on the Cα atoms of the proteins throughout the 900 ns NPT production run for each of the four replicas. Each trace in the graph corresponds to one of the four replicas, depicted in distinct colors: black (Replica 1), red (Replica 2), green (Replica 3), and blue (Replica 4). These traces represent the Rg over the course of the simulation time, providing insight into the protein backbone's compactness.

4.4 Average values of RMSD, RMSF for each protein chain and Rg for protein complexes

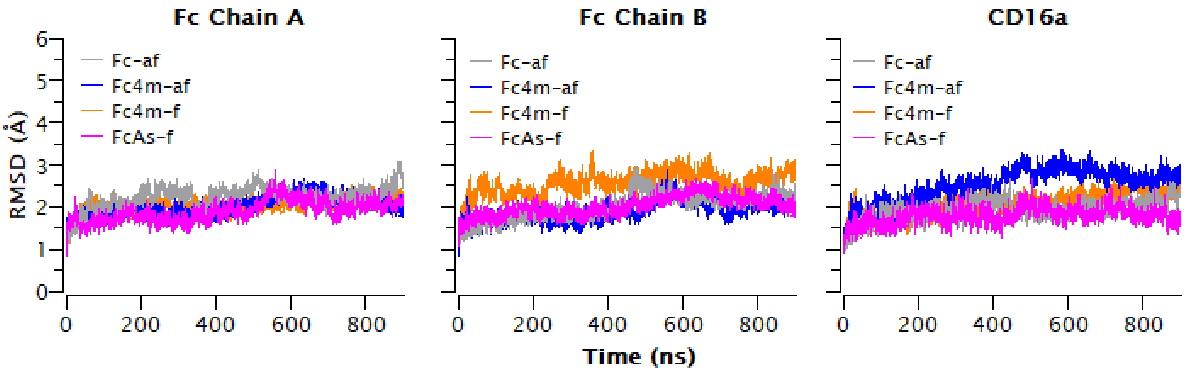

Figure S4a : Average Cα RMSD for Fc chain A, chain B, and CD16a representing the mean of four 900 ns molecular dynamics simulations. The data points for Fc-af are depicted in grey, for Fc4m-af in blue, for Fc4m-f in orange, and for FcAs-f in pink showing the comparative stability across the averaged simulations for each molecular configuration.

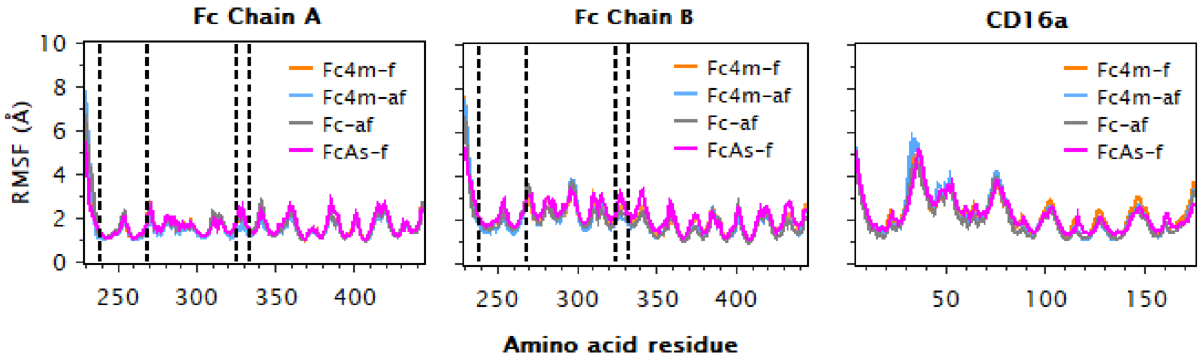

Figure S4b : Average RMSF of amino acid residues in Fc Chain A, Chain B, and CD16a across four 900 ns simulations. Data is represented for protein complexes Fc-af (grey), Fc4m-af (blue), Fc4m-f (orange) and FcAs-f (pink). Four mutation sites in Fc4m-af and Fc4m-f are indicated with dashed lines.

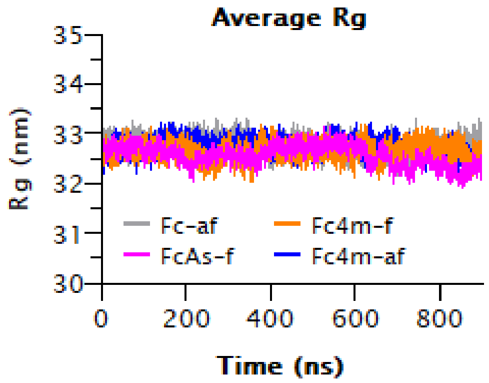

Figure S4c : Average Radius of gyration (*R*g) of the four dynamics of the four complexes, Fc-af, Fc4m-af, Fc4m-f, and FcAs-f.

4.5 Solvent Accessible Surface Area of protein complexes or glycan chains

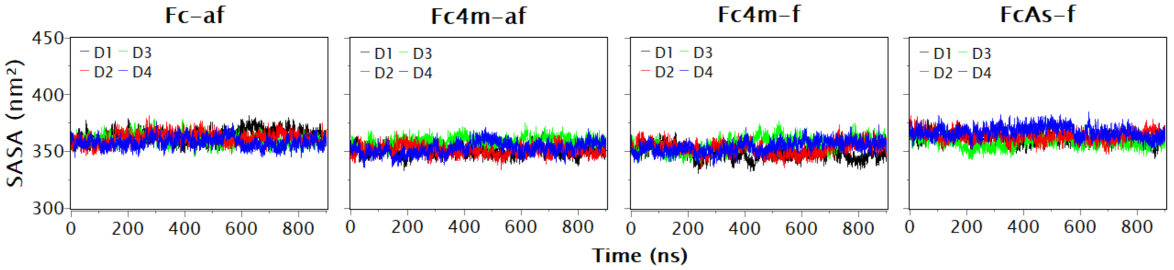

Figure S5a : Solvent Accessible Surface Area (SASA) for the four protein complexes, Fc-af, Fc4m-af, Fc4m-f, and FcAs-f with all glycans analyzed over a 900 ns timeframe at neutral pH. Each panel tracks four simulation replicas (D1 to D4), depicted in black, red, green, and blue respectively.

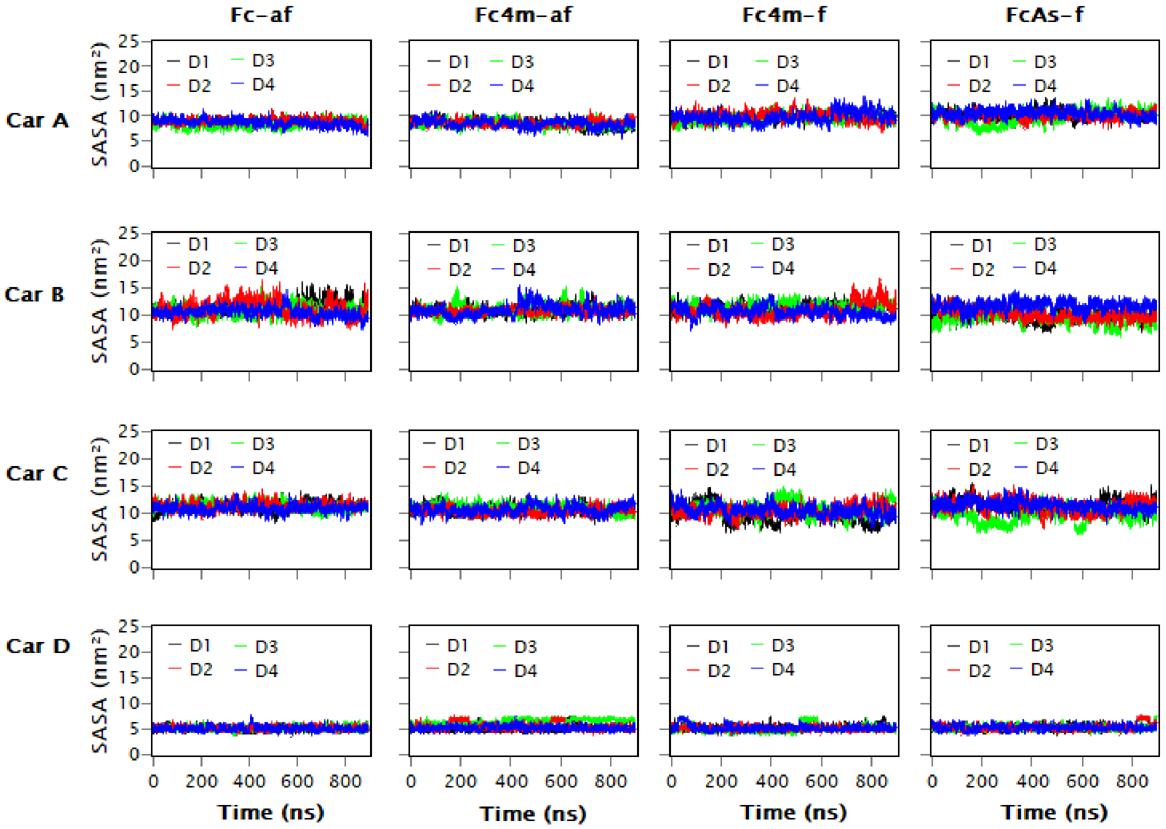

Figure S5b : Solvent Accessible Surface Area (SASA) profiles for glycan chains over 900 ns molecular dynamics simulations. Each row represents a different glycan chain (Fc Chain A, Car A; Fc Chain B, Car B; CD16a, Car C and Car D) with their respective SASA trends shown for Fc-af, Fc4m-af, Fc4m-f, and FcAs-f. Within each plot, four replicas are depicted (D1 to D4).

4.6 Per-atom RMSF of glycan residues across Fc glycovariants

Figure S6: Root-mean-square fluctuations (RMSF, in Å) of the **C1 atom** of each monosaccharide from the four core glycans (CAR A, CAR B on Fc; CAR C, CAR D on CD16a), computed over four independent 900-ns MD replicas (D1 = black, D2 = red, D3 = green, D4 = blue), for each Fc glycovariant: Fc-af, Fc4m-af, Fc4m-f, and FcAs-f.

Each subplot corresponds to one glycan chain and one Fc–CD16a complex, showing per-residue flexibility along the glycan tree. Labels on the x-axis identify the sugar residues. Y-axis values reflect the atomic displacement of the C1 atom over time.

Glycan nomenclature:

- **CAR A** = glycan on Fc chain A
- **CAR B** = glycan on Fc chain B
- **CAR C** and **CAR D** = glycans on CD16a chains

Higher RMSF values indicate greater flexibility of the glycan residue in the given context.

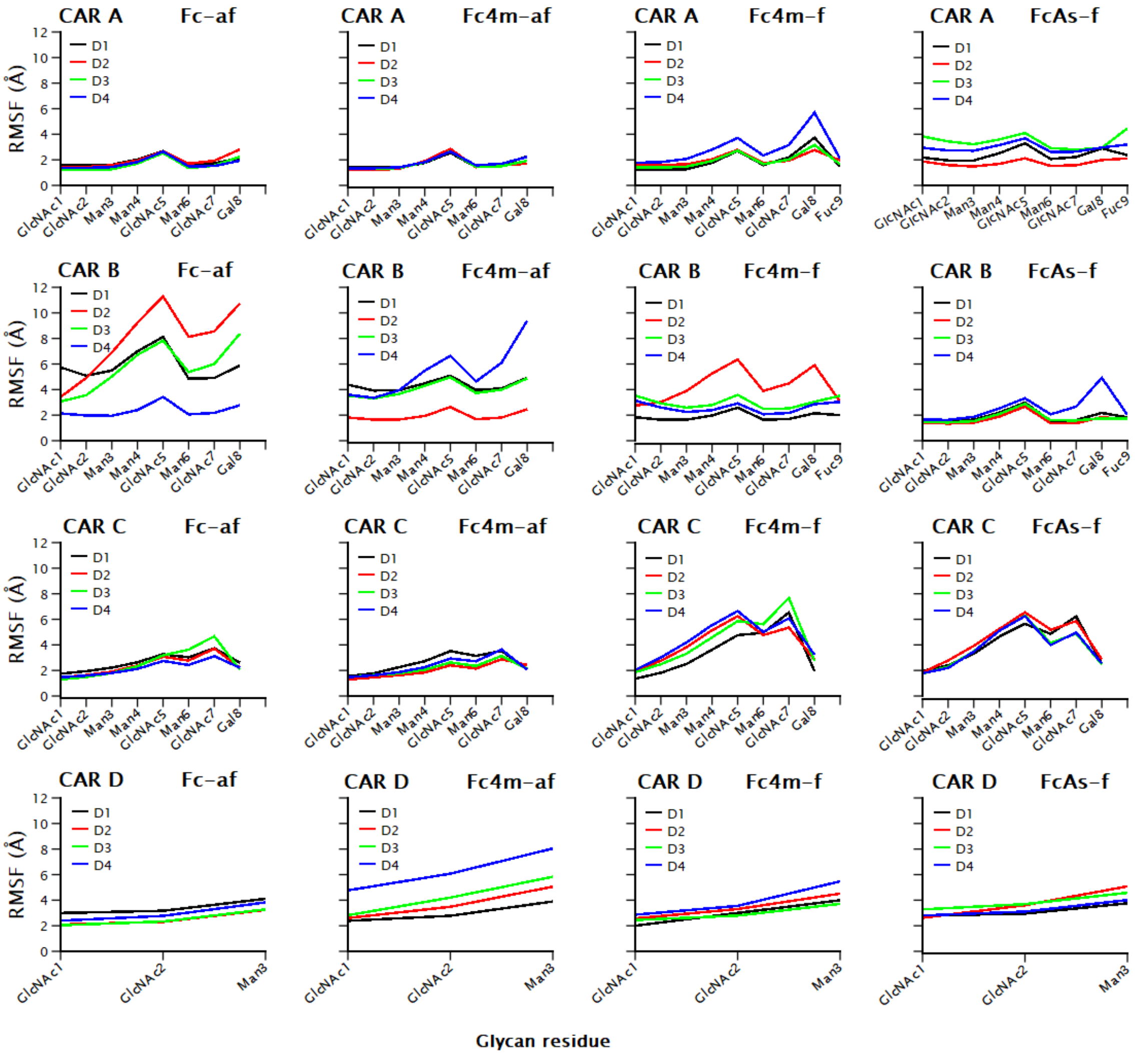

5 Noncovalent bond interactions between amino acids

	Fc-af		Fc4m-af		Fc4m-f		FcAs-f	
**HBOND Interaction (ns)**	Mean	SD	Mean	SD	Mean	SD	Mean	SD
A234TYR-C120LYS							286.3	284.0
A235LEU-C119HIS			202.9	116.3	178.5	108.2	98.5	94.4
A235LEU-C135HIS					94.8	88.8		
A236GLY-C134HIS	530.1	325.9	869.0	21.8	695.0	288.1		
A237GLY-C120LYS	317.0	63.0	129.1	5.3	122.9	21.4	345.3	267.4
A239SER-C120LYS	561.6	31.9						
A265ASP-C120LYS	863.9	17.5						
A265ASP-C132TYR	854.0	41.3	882.4	9.1	799.9	116.2	389.2	223.8
A268HIS-C131LYS	157.8	30.7						
A296TYR-C127GLY	132.4	10.1	93.3	13.7			103.3	44.8
A296TYR-C128LYS	206.6	87.9	224.6	67.3				
A297ASN-C122THR							307.0	280.6
A327ASP-C134HIS							207.5	125.3
A349TYR-B360LYS	274.9	139.3	395.9	73.1	421.1	57.3		
A360LYS-B349TYR	284.7	117.7	362.1	143.7	312.9	70.4	146.8	43.4
A364SER-B370LYS	233.2	76.0	169.1	170.4	169.8	78.1	170.3	147.5
A366THR-B407TYR	893.7	3.6	893.6	2.6	876.6	23.7		
A370LYS-B364SER	284.4	164.7	101.1	70.1	119.9	57.5		
A392LYS-B398LEU							106.4	25.7
A398LEU-B392LYS			106.4	10.6	96.8	17.4		
A407TYR-B366THR	884.1	14.3	893.1	6.8	890.7	2.2	863.4	29.7
B235LEU-C161LYS	125.9	125.6					115.2	140.4
B236GLY-C161LYS	158.5	84.3	141.8	40.9	384.5	292.2		
B237GLY-C161LYS	495.6	145.4	641.0	114.9	717.1	128.1	324.1	185.9
B238PRO-C161LYS	146.7	79.7	112.7	66.7	420.0	362.7	148.1	173.9
B239SER-C161LYS	459.5	152.0					378.5	48.0
B240VAL-C161LYS							151.9	156.3
B330LYS-C88ILE							272.3	92.1
B332GLU-C90TRP			135.6	95.8				

Table S2. Intermolecular Hydrogen bond interactions between amino acids of different protein chains over a 90 ns cutoff of a 900 ns simulation.

Average hydrogen bond interaction durations (in nanoseconds) and standard deviations (SD) for different amino acid pairs for complexes Fc-af, Fc4m-af, Fc4m-f, and FcAs-f. Each pair represents a specific hydrogen bond interaction between residues, with their respective positions in the protein sequence. Fc Chain A and Fc Chain B are noted A and B, respectively, while CD16a is noted as C. No values indicate no significant hydrogen bond detected under the given condition.

IONIC Interaction (ns)	Fc-af		Fc4m-af		Fc4m-f		FcAs-f	
	Mean	SD	Mean	SD	Mean	SD	Mean	SD
A239ASP-C120LYS			884.2	6.9	883.2	5.8		
A265ASP-C120LYS	856.4	18.4	874.3	7.1	888.4	3.5	821.9	77.7
A268ASP-C131LYS							832.8	52.2
A269GLU-C131LYS	463.4	63.9	689.9	28.5	426.4	189.9	789.9	55.9
A356ASP-B439LYS	833.6	31.3	862.0	9.1	839.3	10.1	828.6	22.8
A357GLU-B370LYS	399.3	115.4	238.6	229.8	314.0	173.4	223.9	194.9
A370LYS-B357GLU	441.8	212.8	153.1	109.1	228.5	86.0	856.4	11.6
A392LYS-B399ASP	167.4	150.0			101.1	79.4	117.8	117.6
A399ASP-B409LYS	864.7	21.0	889.5	9.6	872.9	9.9	840.0	49.3
A401ASP-B360LYS							134.2	215.2
A409LYS-B399ASP	874.1	4.5	883.6	10.8	825.4	47.2	881.2	18.0
A439LYS-B356ASP	840.8	13.4	850.8	10.5	843.9	4.6		
B233GLU-C161LYS	175.1	175.2						
B239ASP-C161LYS			847.8	36.9	807.5	86.9		
B265ASP-C161LYS	483.3	161.1	671.7	131.4	326.8	342.9	431.6	276.8
B330LYS-C21GLU							274.6	239.8
B332GLU-C161LYS					103.7	146.0		
B334GLU-C161LYS							153.3	156.6

Table S3. Intermolecular Ionic interactions between charged amino acids of different protein chains over a 90 ns cutoff of a 900 ns simulation.

Mean interaction durations (in nanoseconds) and standard deviations (SD) for various ion pairs for different complexes Fc-af, Fc4m-af, Fc4m-f, and FcAs-f. Each row represents a specific ionic interaction between an aspartate (Asp) or glutamate (Glu) residue and a lysine (Lys) residue, with their respective positions in the protein sequence. Fc Chain A and Fc Chain B are noted A and B, respectively, while CD16a is noted as C. No Arg residues participate in intermolecular ionic interactions. No values indicate no significant interaction observed under the given condition.

Table S4-1. Cross-complex Welch t-tests on Protein–Protein Hydrogen Bonds.

Two-sided Welch's t-test (unequal variances). Durations are per-replica means. Only significant comparisons ($p < 0.05$) are listed. Durations and Δ reported with one decimal place.
Protein 1 / Protein 2: residue identities formatted Chain:ResidueNumber:ResidueName (e.g., A:237:_:GLY). A/B are the two IgG-Fc chains; C is CD16a.
Complex A / Complex B: the two complexes compared (e.g., Fc-af, Fc4m-af, Fc4m-f, FcAs-f).
Mean A (ns) / Mean B (ns): per-replica mean duration within Complex A or Complex B.
Δ (A–B) (ns): mean difference = (Mean A) − (Mean B); positive values favor Complex A.
p-value: Welch t-test p-value (unadjusted), α = 0.05.

Protein 1	Protein 2	Complex A	Complex B	Mean A (ns)	Mean B (ns)	Δ (A–B) (ns)	p-value
**A:237:GLY**	C:120:LYS	Fc-af	Fc4m-af	317.0	129.1	187.9	0.0137
**A:237:GLY**	C:120:LYS	Fc-af	Fc4m-f	317.0	122.9	194.1	0.0089
**A:265:ASP**	C:132:TYR	Fc-af	FcAs-f	854.0	389.2	464.8	0.0346
**A:265:ASP**	C:132:TYR	Fc4m-af	FcAs-f	882.4	389.2	493.2	0.0315
**A:265:ASP**	C:132:TYR	Fc4m-f	FcAs-f	799.9	389.2	410.7	0.0417
**A:296:TYR**	C:127:GLY	Fc-af	Fc4m-af	132.4	93.3	39.1	0.0087
**A:296:TYR**	C:127:GLY	Fc-af	Fc4m-f	132.4	51.5	80.9	0.0032
**A:296:TYR**	C:127:GLY	Fc4m-af	Fc4m-f	93.3	51.5	41.8	0.0341
**A:296:TYR**	C:128:LYS	Fc4m-af	Fc4m-f	224.6	65.9	158.7	0.0253
**A:296:TYR**	C:128:LYS	Fc4m-af	FcAs-f	224.6	78.6	146.0	0.0341
**B:236:GLY**	C:161:LYS	Fc4m-af	FcAs-f	141.8	60.3	81.5	0.0420
**B:237:GLY**	C:161:LYS	Fc4m-f	FcAs-f	717.1	324.1	393.0	0.0273

Table S4-2. Unique Protein–Protein Hydrogen Bonds.

Pairs present in exactly one complex. Mean ± SD (ns) over four replicas. Values rounded to one decimal place. Count: number of replicas (typically 4).

Protein 1	Protein 2	Complex	Mean (ns)	SD (ns)	Count
**A:239:SER**	C:120:LYS	Fc-af	561.6	36.8	4
**A:268:HIS**	C:131:LYS	Fc-af	157.8	35.4	4
**A:234:TYR**	C:120:LYS	FcAs-f	286.3	327.9	4
**A:235:TYR**	C:119:HIS	FcAs-f	98.5	109.0	4
**A:327:ASP**	C:134:HIS	FcAs-f	207.5	144.7	4
**B:240:VAL**	C:161:LYS	FcAs-f	151.9	180.4	4
**B:330:LYS**	C:88:ILE	FcAs-f	272.3	106.4	4
**B:349:TYR**	A:360:LYS	FcAs-f	146.8	50.1	4
**B:366:SER**	A:407:TYR	FcAs-f	863.4	34.3	4
**B:370:LYS**	A:364:SER	FcAs-f	170.3	170.3	4
**B:398:LEU**	A:392:LYS	FcAs-f	106.4	29.6	4

Table S5-1. Cross-complex Welch t-tests on Protein–Protein Ionic Bonds.

Two-sided Welch's t-test (unequal variances). Durations are per-replica means. Only significant comparisons ($p < 0.05$) are listed. Durations and Δ reported with one decimal place.
Protein 1 / Protein 2: residue identities formatted Chain:ResidueNumber:ResidueName. A/B are the two IgG-Fc chains; C is CD16a.
Complex A / Complex B: the two complexes compared (e.g., Fc-af, Fc4m-af, Fc4m-f, FcAs-f).
Mean A (ns) / Mean B (ns): per-replica mean duration within Complex A or Complex B.
Δ (A–B) (ns): mean difference = (Mean A) − (Mean B); positive values favor Complex A.
p-value: Welch t-test p-value (unadjusted), $\alpha = 0.05$.

Protein 1	Protein 2	Complex A	Complex B	Mean A (ns)	Mean B (ns)	Δ (A–B) (ns)	p-value
**A:265:ASP**	C:120:LYS	Fc4m-af	Fc4m-f	874.3	888.4	-14.1	0.0328
**A:269:GLU**	C:131:LYS	Fc-af	Fc4m-af	463.4	689.9	-226.5	0.0045
**A:269:GLU**	C:131:LYS	Fc-af	FcAs-f	463.4	789.9	-326.5	0.0006
**A:269:GLU**	C:131:LYS	Fc4m-af	FcAs-f	689.9	789.9	-100.0	0.0450
**A:269:GLU**	C:131:LYS	Fc4m-f	FcAs-f	426.4	789.9	-363.5	0.0402
**A:356:ASP**	B:439:LYS	Fc4m-af	Fc4m-f	862.0	839.3	22.7	0.0278

Table S5-2. Unique Protein–Protein Ionic Bonds.

Pairs present in exactly one complex. Mean ± SD (ns) over four replicas. Values rounded to one decimal place. Count: number of replicas (typically 4).

Protein 1	Protein 2	Complex	Mean (ns)	SD (ns)	Count
**B:233:GLU**	C:161:LYS	Fc-af	175.1	202.3	4
**A:268:ASP**	C:131:LYS	FcAs-f	832.8	60.3	4
**B:330:LYS**	C:21:GLU	FcAs-f	274.6	276.9	4
**B:334:GLU**	C:161:LYS	FcAs-f	153.3	180.8	4
**B:357:GLU**	A:370:LYS	FcAs-f	856.4	13.4	4
**B:360:LYS**	A:401:ASP	FcAs-f	134.2	248.4	4
**B:370:LYS**	A:357:GLU	FcAs-f	223.9	225.0	4
**B:399:ASP**	A:392:LYS	FcAs-f	117.8	135.8	4
**B:399:ASP**	A:409:LYS	FcAs-f	881.2	20.8	4
**B:409:LYS**	A:399:ASP	FcAs-f	840.0	56.9	4
**B:439:LYS**	A:356:ASP	FcAs-f	828.6	26.4	4

6 Intermolecular van der Waals interaction

A)

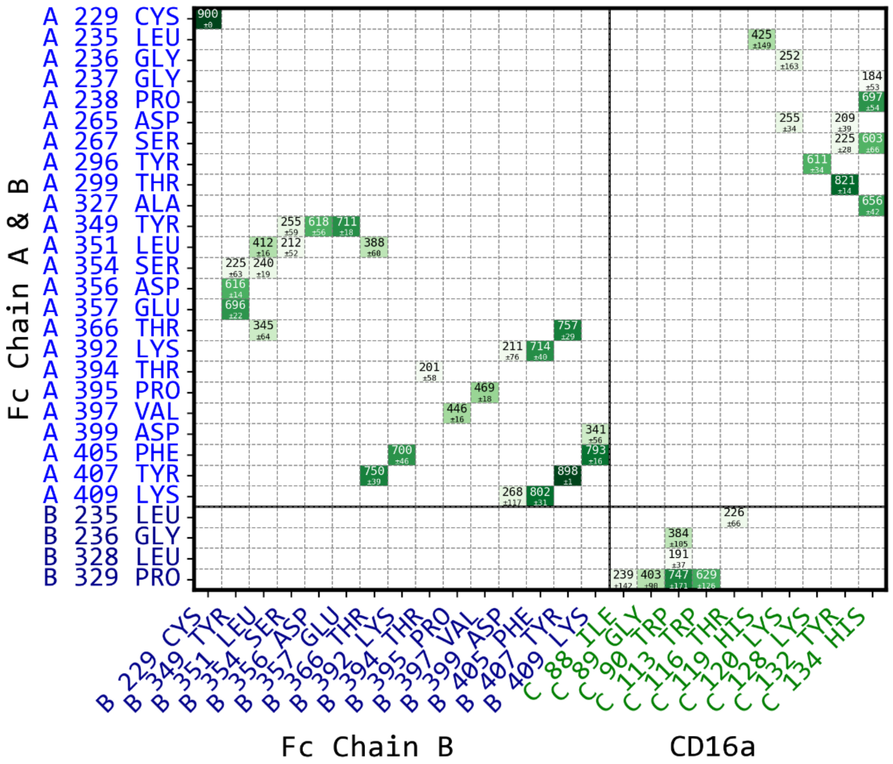

B)

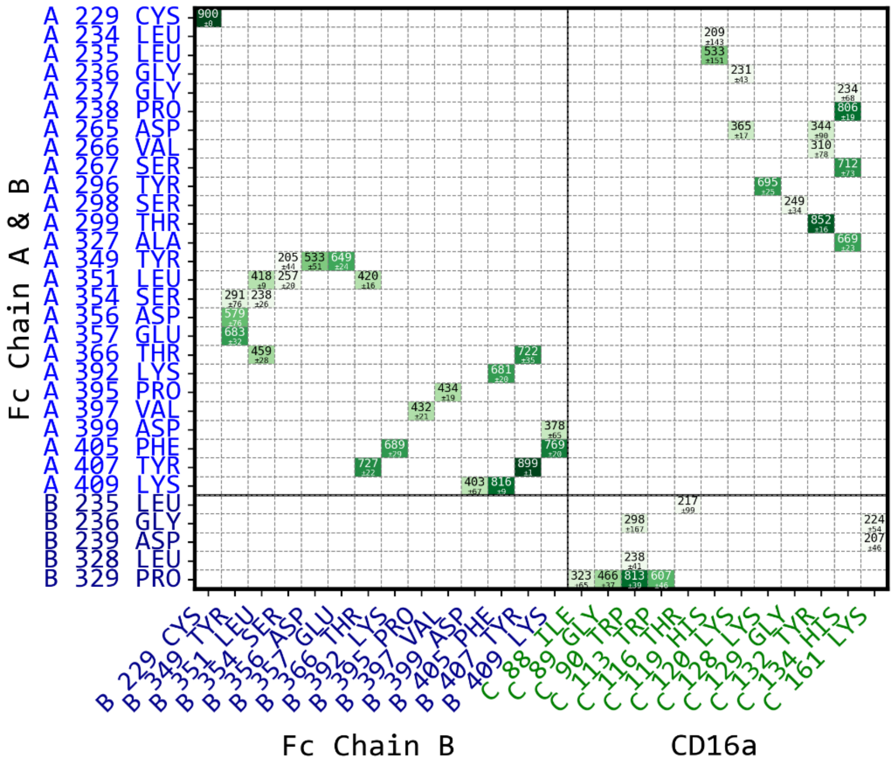

C)

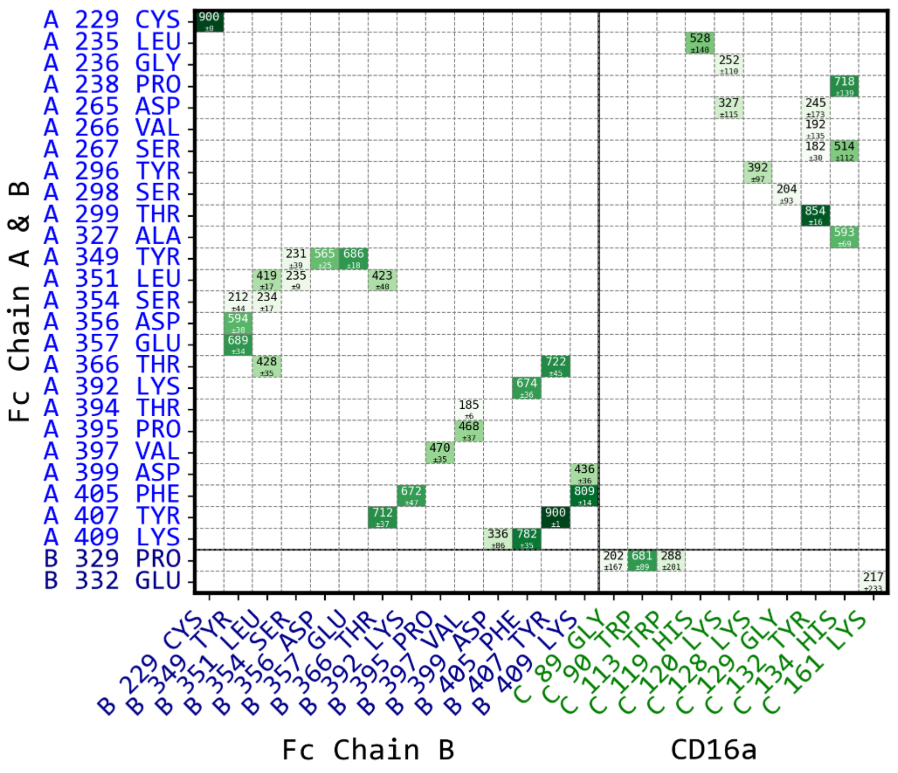
A 229 CYS
A 235 LEU
A 236 GLY
A 238 PRO
A 265 ASP
A 266 VAL
A 267 SER
A 296 TYR
A 298 SER
A 299 THR
A 327 ALA
A 349 TYR
A 351 LEU
A 354 SER
A 356 ASP
A 357 GLU
A 366 THR
A 392 LYS
A 394 THR
A 395 PRO
A 397 VAL
A 399 ASP
A 405 PHE
A 407 TYR
A 409 LYS
B 329 PRO
B 332 GLU
Fc Chain A & B
B 229 CYS
B 349 TYR
B 351 LEU
B 354 SER
B 356 ASP
B 357 GLU
B 366 THR
B 392 LYS
B 395 PRO
B 397 VAL
B 399 ASP
B 405 PHE
B 407 TYR
B 409 LYS
C 89 GLY
C 90 TRP
C 113 TRP
C 119 HIS
C 120 LYS
C 128 LYS
C 129 GLY
C 132 TYR
C 134 HIS
C 161 LYS
Fc Chain B
CD16a

D)

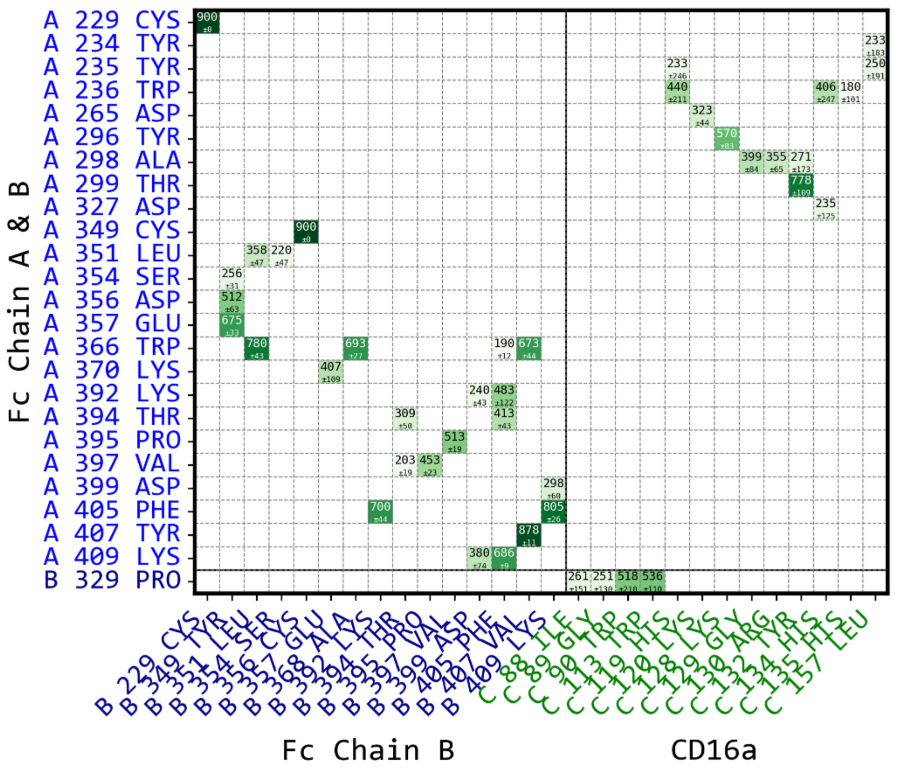
A 229 CYS
A 234 TYR
A 235 TYR
A 236 TRP
A 265 ASP
A 296 TYR
A 298 ALA
A 299 THR
A 327 ASP
A 349 CYS
A 351 LEU
A 354 SER
A 356 ASP
A 357 GLU
A 366 TRP
A 370 LYS
A 392 LYS
A 394 THR
A 395 PRO
A 397 VAL
A 399 ASP
A 405 PHE
A 407 TYR
A 409 LYS
B 329 PRO
Fc Chain A & B
Fc Chain B
CD16a

Figure S7 : Heatmap representing intermolecular Van der Waals interaction durations.

The heatmap shows the average durations of Van der Waals interactions over a 900 ns calculation within the Fc (Chains A & B) and CD16a on four distinct complexes: Fc-af (A), Fc4m-af (B), Fc4m-f (C) and FcAs-f (D). Each cell in the heatmap represents the mean duration between a pair of residues from different protein chains, annotated with the standard deviation based on four independent molecular dynamics simulations for each complex. The color gradient from white to green indicates increasing interaction duration. A cutoff of 180 ns has been applied to the visualization of interactions.

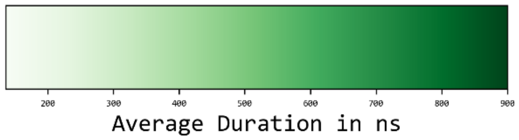

Table S6-1. Cross-complex Welch t-tests on Protein–Protein vdW interactions.

Two-sided Welch's t-test (unequal variances). Durations are per-replica means. Only significant comparisons ($p < 0.05$) are listed. Durations and Δ reported with one decimal place.
Protein 1 / Protein 2: residue identities formatted Chain:ResidueNumber:ResidueName. A/B are the two IgG-Fc chains; C is CD16a.
Complex A / Complex B: the two complexes compared (e.g., Fc-af, Fc4m-af, Fc4m-f, FcAs-f).
Mean A (ns) / Mean B (ns): per-replica mean duration within Complex A or Complex B.
Δ (A–B) (ns): mean difference = (Mean A) − (Mean B); positive values favor Complex A.
p-value: Welch t-test p-value (unadjusted), $\alpha = 0.05$.

Protein 1	Protein 2	Complex A	Complex B	Mean A (ns)	Mean B (ns)	Δ (A–B) (ns)	p-value
**A:238:PRO**	C:134:HIS	Fc-af	FcAs-f	697.1	167.9	529.1	0.0274
**A:267:SER**	C:134:HIS	Fc-af	FcAs-f	602.6	132.5	470.1	0.0304
**A:267:SER**	C:152:HIS	Fc4m-af	Fc4m-f	711.7	514.5	197.2	0.0492
**A:296:TYR**	C:146:LYS	Fc4m-af	Fc4m-f	695.2	392.4	302.8	0.0097
**A:349:TYR**	B:357:GLU	Fc-af	Fc4m-af	711.2	649.2	62.0	0.0126
**A:366:THR**	B:351:LEU	Fc-af	Fc4m-af	344.6	459.4	-114.8	0.0448
**A:405:PHE**	B:409:LYS	Fc4m-af	Fc4m-f	769.0	808.9	-39.9	0.0335
**A:407:TYR**	B:407:TYR	Fc-af	Fc4m-f	898.3	899.5	-1.2	0.0391
**B:235:LEU**	C:116:THR	Fc-af	FcAs-f	225.7	103.7	122.0	0.0412
**B:239:ASP**	C:179:LYS	Fc4m-af	Fc4m-f	206.9	72.1	134.8	0.0101

Table S6-2. Unique Protein–Protein vdW interactions.

Pairs present in exactly one complex. Mean ± SD (ns) over four replicas. Values rounded to one decimal place. Count: number of replicas (typically 4).

Protein 1	Protein 2	Complex	Mean (ns)	SD (ns)	Count
**A:235:LEU**	C:119:HIS	Fc-af	425.2	171.7	4
**A:236:GLY**	C:120:LYS	Fc-af	252.4	188.1	4
**A:327:ALA**	C:134:HIS	Fc-af	655.7	49.1	4
**A:234:TYR**	C:157:LEU	FcAs-f	233.1	211.1	4
**A:235:TYR**	C:119:HIS	FcAs-f	232.8	283.6	4
**A:235:TYR**	C:157:LEU	FcAs-f	250.1	220.9	4
**A:236:TRP**	C:119:HIS	FcAs-f	440.1	243.5	4
**A:236:TRP**	C:134:HIS	FcAs-f	405.6	284.7	4
**A:236:TRP**	C:135:HIS	FcAs-f	180.2	117.1	4
**A:298:ALA**	C:129:GLY	FcAs-f	399.0	96.4	4
**A:298:ALA**	C:130:ARG	FcAs-f	354.8	74.7	4
**A:298:ALA**	C:132:TYR	FcAs-f	271.1	199.5	4
**A:327:ASP**	C:134:HIS	FcAs-f	235.0	143.8	4
**B:229:CYS**	A:229:CYS	FcAs-f	900.0	0.0	4
**B:349:TYR**	A:354:SER	FcAs-f	255.9	36.1	4
**B:349:TYR**	A:356:ASP	FcAs-f	512.2	72.7	4
**B:349:TYR**	A:357:GLU	FcAs-f	674.8	38.6	4
**B:351:LEU**	A:351:LEU	FcAs-f	357.5	53.9	4
**B:351:LEU**	A:366:TRP	FcAs-f	779.6	49.7	4
**B:354:SER**	A:351:LEU	FcAs-f	220.2	53.9	4
**B:356:CYS**	A:349:CYS	FcAs-f	900.0	0.0	4
**B:357:GLU**	A:370:LYS	FcAs-f	407.5	125.8	4
**B:368:ALA**	A:366:TRP	FcAs-f	692.8	31.5	4
**B:392:LYS**	A:405:PHE	FcAs-f	699.7	50.8	4
**B:394:THR**	A:394:THR	FcAs-f	308.9	57.8	4
**B:394:THR**	A:397:VAL	FcAs-f	203.0	22.2	4
**B:395:PRO**	A:397:VAL	FcAs-f	453.3	26.8	4
**B:397:VAL**	A:395:PRO	FcAs-f	512.7	21.5	4
**B:399:ASP**	A:392:LYS	FcAs-f	239.6	50.1	4
**B:399:ASP**	A:409:LYS	FcAs-f	380.5	85.8	4
**B:405:PHE**	A:366:TRP	FcAs-f	189.6	13.3	4
**B:405:PHE**	A:392:LYS	FcAs-f	482.7	140.7	4
**B:405:PHE**	A:394:THR	FcAs-f	413.1	49.5	4
**B:405:PHE**	A:409:LYS	FcAs-f	686.0	10.4	4
**B:407:VAL**	A:366:TRP	FcAs-f	672.5	50.8	4
**B:407:VAL**	A:407:TYR	FcAs-f	878.2	12.2	4
**B:409:LYS**	A:399:ASP	FcAs-f	298.3	69.6	4
**B:409:LYS**	A:405:PHE	FcAs-f	804.6	29.9	4

7 Hydrogen Bond Interaction Between Protein Residues and Glycan Sugars

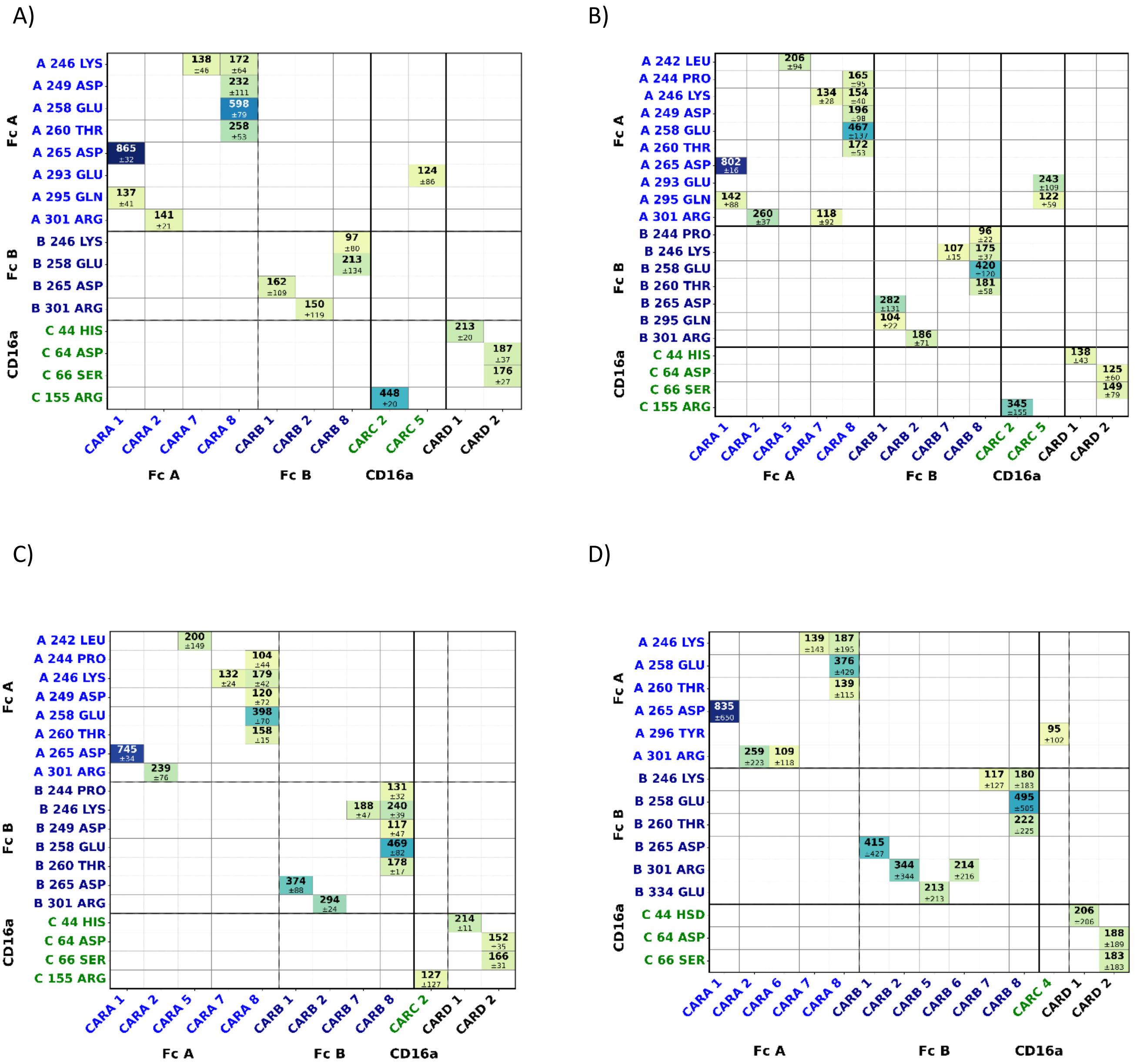

Figure S8 : Heatmap of Hydrogen Bond Interaction Durations Between Protein Residues and Glycan Sugars.

This heatmap illustrates the average durations of hydrogen bond interactions between residues on protein chains (Fc: chains A and B; CD16a: chain C) and glycan sugars, over a 900 ns molecular dynamics simulation. Four distinct complexes are analyzed: Fc-af (A), Fc4m-af (B), Fc4m-f (C), and FcAs-f (D). Each cell represents the mean interaction duration between a protein residue and a glycan sugar, annotated with the standard deviation derived from four independent simulations per complex. Interactions involving residues and glycans that belong to the same protein chain (intrachain) as well as interactions between different chains (interchain) are included. The color gradient ranges from yellow (short-lived interactions) to blue (long-lived interactions), with only interactions exceeding 90 ns on average shown.

CAR A & CAR B 1 GlcNAc, 2 GlcNAc, 3 Man, 4 Man, 5 GlcNAc, 6 Man, 7 GlcNAc, 8 Gal 9 Fuc
CAR C : 1 GlcNAc, 2 GlcNAc, 3 Man, 4 Man, 5 GlcNAc, 6 Man, 7 GlcNAc 8 Fuc
CAR D : 1 GlcNAc, 2 GlcNAc, 3 Man

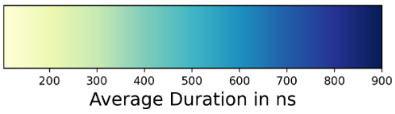

Table S8-1. Cross-complex Welch t-tests on Protein–Glycan Hydrogen Bonds.

Two-sided Welch's t-test (unequal variances). Durations are per-replica means. Only significant comparisons ($p < 0.05$) are listed. Durations and Δ reported with one decimal place.
Protein: Residue from proteins (chains A, B, C), written as Chain ResidueNumber ResidueName (e.g., A 258 GLU = chain A, residue 258, glutamate).
Glycan: Residue from the N-glycans of Fc (CARA, CARB) or CD16a (CARC, CARD), written as Chain ResidueNumber SugarType (e.g., CARA 8 BGAL = β-galactose #8 on Fc chain A).
Complex A / B: Fc glycovariants compared (Fc-af, Fc4m-af, Fc4m-f, FcAs-f).
Mean A / B: Mean duration (in ns) of the interaction for each complex (averaged over 4 MD replicas).
Δ (A–B): Duration difference between the two complexes.
p-value: Welch's test p-value (significance at $\alpha = 0.05$).

Protein	Glycan	Complex A	Complex B	Mean A (ns)	Mean B (ns)	Δ (A–B) (ns)	p-value
**A 258 GLU**	CARA 8 BGAL	Fc-af	Fc4m-f	598.1	398.3	199.9	0.0171
**A 260 THR**	CARA 8 BGAL	Fc-af	Fc4m-f	258.0	157.6	100.5	0.0415
**A 265 ASP**	CARA 1 BGLCNA	Fc-af	Fc4m-af	865.4	802.3	63.1	0.0338
**A 265 ASP**	CARA 1 BGLCNA	Fc-af	Fc4m-f	865.4	744.9	120.5	0.0045
**A 293 GLU**	CARC 5 BGLCNA	Fc4m-af	FcAs-f	243.4	0.4	243.0	0.0308
**A 295 GLN**	CARC 5 BGLCNA	Fc4m-af	FcAs-f	122.0	0.1	121.9	0.0375
**A 301 ARG**	CARA 2 BGLCNA	Fc-af	Fc4m-af	141.0	259.9	-118.9	0.0054
**B 244**	CARB 8 BGAL	Fc-af	Fc4m-af	27.2	95.9	-68.7	0.0232
**B 244**	CARB 8 BGAL	Fc-af	Fc4m-f	27.2	131.1	-103.9	0.0067
**B 246 LYS**	CARB 7 BGLCNA	Fc-af	Fc4m-f	60.2	187.8	-127.6	0.0154
**B 246 LYS**	CARB 8 BGAL	Fc-af	Fc4m-f	97.4	239.7	-142.3	0.0452
**B 249 ASP**	CARB 8 BGAL	Fc-af	Fc4m-f	31.5	116.9	-85.4	0.0436
**B 258 GLU**	CARB 8 BGAL	Fc-af	Fc4m-f	212.8	469.3	-256.5	0.0370
**B 260 THR**	CARB 8 BGAL	Fc-af	Fc4m-f	82.5	177.6	-95.1	0.0401
**B 265 ASP**	CARB 1 BGLCNA	Fc-af	Fc4m-f	162.5	373.9	-211.4	0.0413
**C 155 ARG**	CARC 2 BGLCNA	Fc-af	Fc4m-f	448.0	126.7	321.3	0.0204
**C 155 ARG**	CARC 2 BGLCNA	Fc-af	FcAs-f	448.0	40.4	407.6	0.0001
**C 155 ARG**	CARC 2 BGLCNA	Fc4m-af	FcAs-f	345.1	40.4	304.7	0.0379
**C 44 HIS**	CARD 1 BGLCNA	Fc-af	Fc4m-af	213.4	137.8	75.7	0.0482

Table S8-2. Unique Protein–Glycan Hydrogen Bonds.

Pairs present in exactly one complex. Mean ± SD (ns) over four replicas. Values rounded to one decimal place. Count: number of replicas (typically 4).

Protein	Glycan	Complex	Mean (ns)	SD (ns)	Count
**A 246 LYS**	CARB 7 BGLCNA	FcAs-f	139.4	165.5	4
**A 246 LYS**	CARB 8 BGAL	FcAs-f	187.3	225.1	4
**A 258 GLU**	CARB 8 BGAL	FcAs-f	375.7	495.8	4
**A 260 THR**	CARB 8 BGAL	FcAs-f	139.3	133.2	4
**A 265 ASP**	CARB 1 BGLCNA	FcAs-f	835.3	750.2	4
**A 301 ARG**	CARB 2 BGLCNA	FcAs-f	259.3	257.6	4
**A 301 ARG**	CARB 6 AMAN	FcAs-f	109.5	136.5	4
**B 246 LYS**	CARA 7 BGLCNA	FcAs-f	117.2	147.1	4
**B 246 LYS**	CARA 8 BGAL	FcAs-f	179.7	211.9	4
**B 258 GLU**	CARA 8 BGAL	FcAs-f	494.8	583.0	4
**B 260 THR**	CARA 8 BGAL	FcAs-f	221.7	260.3	4
**B 265 ASP**	CARA 1 BGLCNA	FcAs-f	415.0	493.3	4
**B 301 ARG**	CARA 2 BGLCNA	FcAs-f	343.8	397.4	4
**B 301 ARG**	CARA 6 AMAN	FcAs-f	214.5	249.3	4
**B 334 GLU**	CARA 5 BGLCNA	FcAs-f	213.2	246.4	4
**C 44 HSD**	CARD 1 BGLCNA	FcAs-f	206.1	238.3	4

In addition, a complementary presence/absence analysis applying the same 90 ns cutoff across all complexes showed that only a limited subset of protein–glycan pairs are long-lived in one glycovariant and absent (or below cutoff) in another; for example, in Fc4m-af, Glu293 and Gln295 of Fc chain A form hydrogen bonds with the CD16a CARC 5 GlcNAc that are not detected above this threshold in Fc4m-f or FcAs-f.

8 Hydrogen bond interaction between sugars of glycan chains.

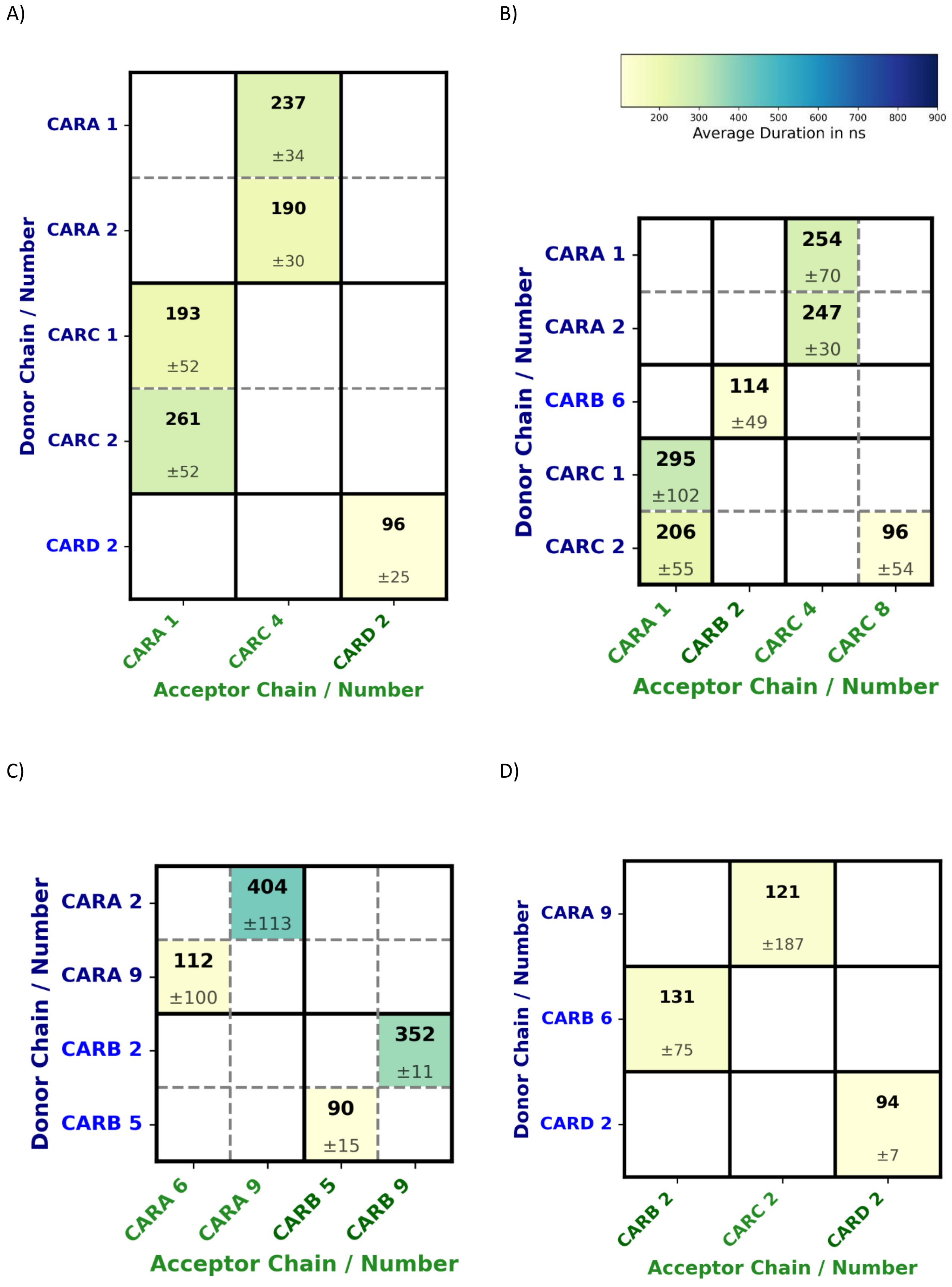

Figure S9 : Heatmap representing hydrogen bond interaction durations between sugars of glycan chains.

The heatmap displays the average durations of intra- and intermolecular hydrogen bond interactions over a 900 ns calculation within the sugars of glycan chains CARA, CARB (on Fc chains A and B), and CARC, CARD (on CD16a), across four distinct complexes: Fc-af (A), Fc4m-af (B), Fc4m-f (C), and FcAs-f (D). Each cell corresponds to the mean duration of hydrogen bonds between two specific glycan sugars, annotated with the standard deviation obtained from four independent molecular dynamics simulations. The color gradient (yellow to blue) indicates increasing interaction durations, with only interactions persisting for more than 90 ns shown for clarity.

CAR A & CAR B 1 GlcNAc, 2 GlcNAc, 3 Man, 4 Man, 5 GlcNAc, 6 Man, 7 GlcNAc, 8 Gal 9 Fuc

CAR C : 1 GlcNAc, 2 GlcNAc, 3 Man, 4 Man, 5 GlcNAc, 6 Man, 7 GlcNAc 8 Fuc

CAR D : 1 GlcNAc, 2 GlcNAc, 3 Man

Table S9-1. Cross-complex Welch t-tests on Glycan–Glycan Hydrogen Bonds.

Two-sided Welch's t-test (unequal variances). Durations are per-replica means. Only significant comparisons ($p < 0.05$) are listed. Durations and Δ reported with one decimal place.
Glycan 1 / Glycan 2: Sugars from the N-glycans of Fc (CARA on Fc chain A; CARB on Fc chain B) or CD16a (CARC, CARD), written as Carrier Index SugarType (e.g., CARA 2 BGLCNA = β-GlcNAc #2 on Fc-A; CARC 4 AMAN = α-mannose #4 on CD16a).
Complex A / B: Fc glycovariants compared (Fc-af, Fc4m-af, Fc4m-f, FcAs-f).
Mean A / B: Mean interaction duration (ns) for each complex (average over 4 MD replicas of 900 ns each).
Δ (A–B): Duration difference between the two complexes.
p-value: Welch's test p-value (significance at $\alpha = 0.05$).

Glycan 1	Glycan 2	Complex A	Complex B	Mean A (ns)	Mean B (ns)	Δ (A–B) (ns)	p-value
**CARA 2 BGLCNA**	CARC 4 AMAN	Fc-af	Fc4m-f	190.4	0.1	190.3	0.0010
**CARA 2 BGLCNA**	CARC 4 AMAN	Fc-af	Fc4m-af	190.4	247.5	-57.1	0.0356
**CARA 2 BGLCNA**	CARC 4 AMAN	Fc4m-f	Fc4m-af	0.1	247.5	-247.4	0.0005
**CARC 1 BGLCNA**	CARA 1 BGLCNA	Fc4m-f	Fc4m-af	81.7	295.1	-213.4	0.0284
**CARC 2 BGLCNA**	CARA 1 BGLCNA	Fc-af	Fc4m-f	261.3	1.5	259.8	0.0021
**CARC 2 BGLCNA**	CARA 1 BGLCNA	Fc4m-f	Fc4m-af	1.5	205.6	-204.1	0.0049

Table S9-2. Unique Glycan–Glycan Hydrogen Bonds.

Pairs present in exactly one complex. Mean ± SD (ns) over four replicas. Values rounded to one decimal place. Count: number of replicas (typically 4).

Glycan 1	Glycan 2	Complex	Mean (ns)	SD (ns)	Count
**CARA 2 BGLCNA**	CARA 9 AFUC	Fc4m-f	404.4	113.1	4
**CARB 2 BGLCNA**	CARB 9 AFUC	Fc4m-f	351.6	11.3	4
**CARA 9 AFUC**	CARA 6 AMAN	Fc4m-f	111.8	100.3	4
**CARA 9 BFUC**	CARC 2 BGLCNA	FcAs-f	120.9	187.0	4

9 Intermolecular Interactions at the CH3–CH3 Interface of Fc Variants

A)

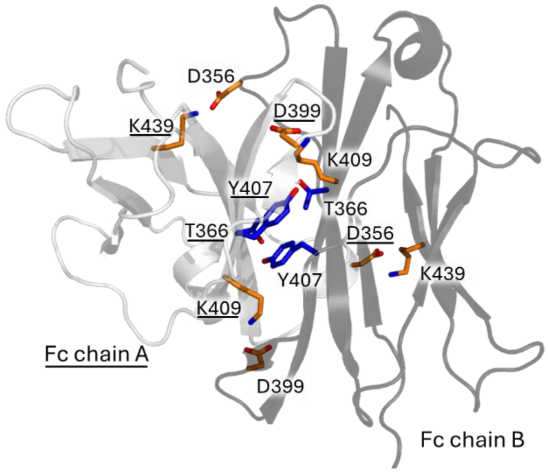

B)

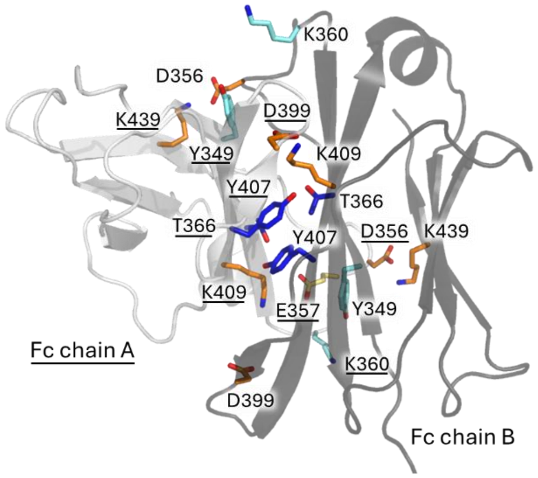

C)

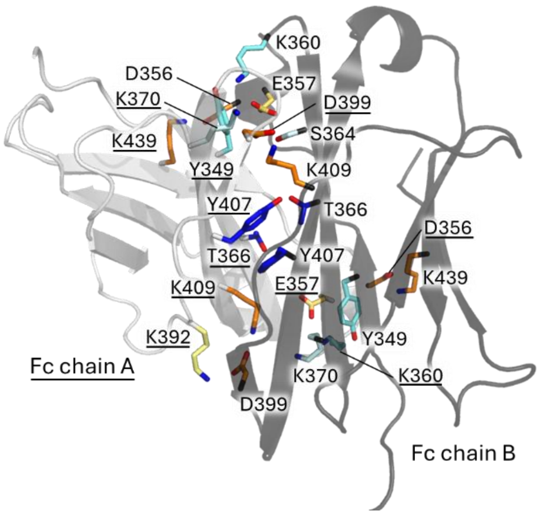

D)

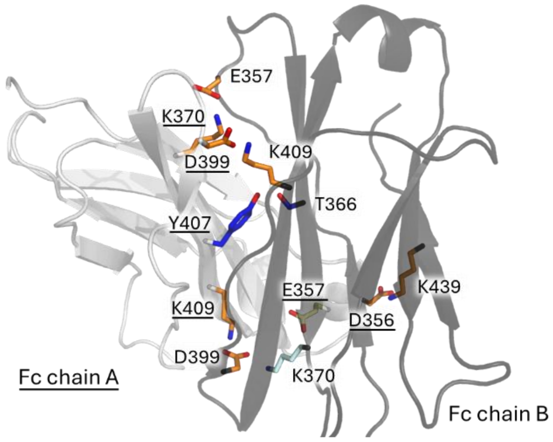

Figure S10 : Residues involved in intermolecular interactions between Fc chains A and B at the CH3–CH3 interface are shown for each complex: Fc-af (A), Fc4m-af (B), Fc4m-f (C), and FcAs-f (D). Fc chain A is shown in light gray, Fc chain B in dark gray. Residues forming hydrogen bonds (blue) or ionic interactions (orange) are represented when their cumulative interaction duration exceeds 200 ns during molecular dynamics simulations. This threshold highlights the most persistent contacts for each complex.

10 The local hydrophobic patch with Trp236 in the FcAs-f complex

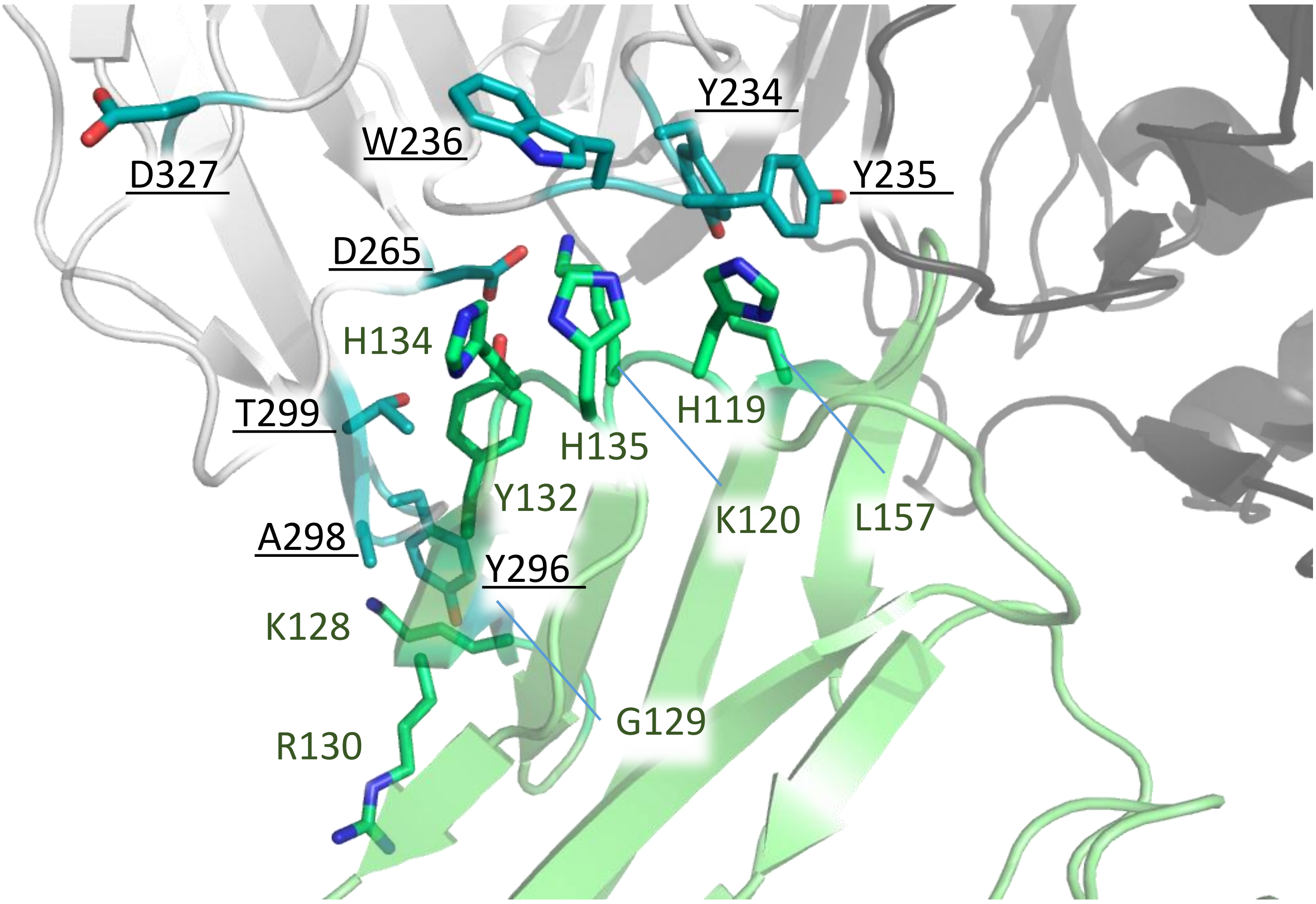

Figure S11 : Persistent hydrophobic interactions at the FcA–CD16a interface in the FcAs-f complex.

Residues around Trp236 involved in hydrophobic interactions between Fc chain A (light gray) and CD16a (green) are shown. Hydrophobic contacts are defined by a cumulative interaction duration exceeding 200 ns over the course of the molecular dynamics simulations, thus highlighting the most persistent nonpolar contacts at the interface. Amino acid labels are underlined in black for FcA residues but not for CD16a residues. The final simulation frame is depicted for illustrative purposes.

11 Pairwise MM/GBSA residue interaction enthalpies for the Fc–CD16a complexes

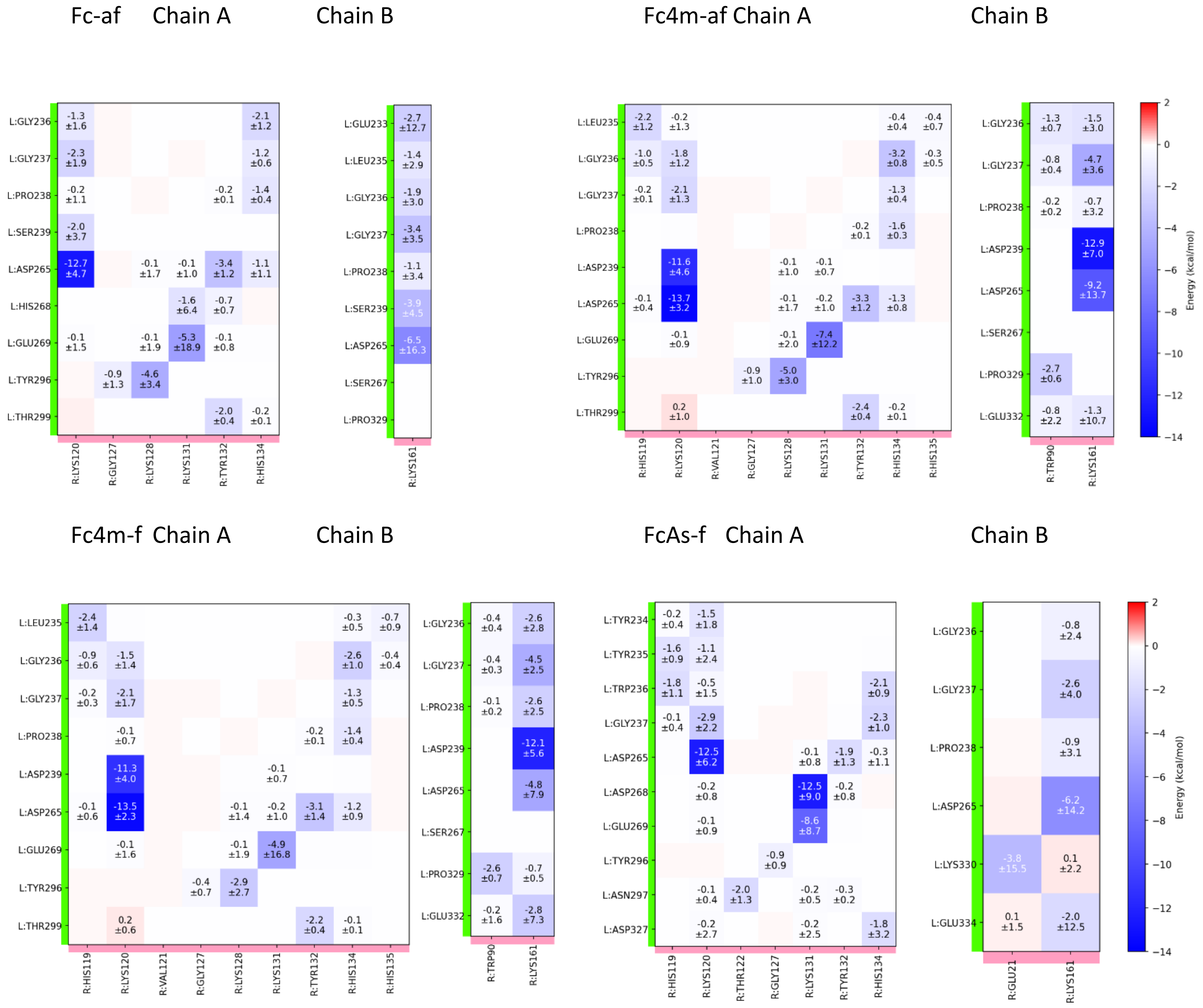

Figure S12 : Pairwise interaction enthalpies (ΔH, kcal·mol^{-1}) between Fc residues (chains A and B; rows) and CD16a residues (columns) computed by MM/GBSA over the 100–1000 ns window of the 1 µs trajectories. A generalized Born implicit solvent model and residue-pair decomposition (decomp = 4) were used, with FcA, FcB and CD16a defined as the solute groups. Ninety-one snapshots per complex were extracted at 10 ns intervals (frames 500–5000). Values shown correspond to the relative enthalpic contributions (molecular mechanics + solvation); entropic terms were not included. Only interactions with $|\Delta H| \geq 0.1$ kcal·mol^{-1} are displayed; empty cells indicate contributions below this threshold. Heatmaps are presented separately for Fc chain A and chain B (L) for each complex: Fc-af, Fc4m-af, Fc4m-f, and FcAs-f. CD16a residues are labeled with the prefix R in all heatmaps.
